# Isozyme-specific comprehensive characterization of transglutaminase-crosslinked substrates in kidney fibrosis

**DOI:** 10.1038/s41598-018-25674-4

**Published:** 2018-05-09

**Authors:** Hideki Tatsukawa, Risa Otsu, Yuji Tani, Ryosuke Wakita, Kiyotaka Hitomi

**Affiliations:** Cellular Biochemistry Lab., Graduate School of Pharmaceutical Sciences, Nagoya University, Furo-cho, Chikusa, Nagoya, 464-8601 Japan

## Abstract

Chronic kidney disease is characterized by prolonged decline in renal function, excessive accumulation of ECM, and progressive tissue fibrosis. Transglutaminase (TG) is a crosslinking enzyme that catalyzes the formation of covalent bonds between glutamine and lysine residues, and is involved in the induction of renal fibrosis via the stabilization of ECM and the activation of TGF-β1. Despite the accumulating evidences indicating that TG2 is a key enzyme in fibrosis, genetic knockout of TG2 reduced by only 50% the elevated protein crosslinking and fibrous protein in renal fibrosis model, whereas treatment with TG inhibitor almost completely reduced these levels. Here, we also clarified the distributions of TG isozymes and their *in situ* activities and identified the isozyme-specific crosslinked substrates for both TG1 and TG2 in fibrotic kidney. We found that TG1 activity was markedly enhanced in renal tubular epithelium and interstitial areas, whereas TG2 activity increased only in the extracellular space. In total, 47 and 67 possible candidates were identified as TG1 and TG2 substrates, respectively, only in fibrotic kidney. Among them, several possible substrates related to renal disease and fibrosis were identified. These findings provide novel insights into the mechanisms of renal fibrosis through the targeting of isozyme-specific TG substrates.

## Introduction

Chronic kidney disease (CKD), including diabetic nephropathy and glomerulonephritis, is characterized by tubulointerstitial fibrosis and glomerulosclerosis that result from progressive remodeling processes, including excessive accumulation of extracellular matrix proteins (ECM), fibroblast proliferation and tubular atrophy^1^. The development of tubulointerstitial scarring is attributed to the expansion of both the tubular basement membrane and the interstitial ECM^2^. Tubular epithelial cells are considered to be significant contributors to the expanding matrix, responding to growth factors such as TGF-β1 by altering ECM metabolism^2,3^. The balance between increased tubular ECM synthesis and reduced ECM breakdown has been implicated in this process.

In the recent years, transglutaminase (TG)-mediated crosslinking reactions have been demonstrated to be crucial to TGF-β1 activation^4,5^, cellular viability^6,7^ and ECM stabilization against proteases^5,8,9^, which underlie these fibrotic remodeling processes. TGs are crosslinking enzymes that catalyze the post-translational modification of substrate proteins predominantly via crosslinking between glutamine (Gln) and either lysine (Lys) residues or a primary amino group such as polyamine in a Ca^2+^-dependent manner^10–14^. In mammalian TG family, the eight TG isozymes, designated factor XIIIa and TG1–7, are widely distributed in whole body and involved in signal transduction, cell adhesion, and membrane integrity. The posttranslational modification by TG family are essential for multiple biological processes such as transcriptional regulation, blood coagulation, skin barrier formation, and extracellular matrix assembly but can also contribute to the pathophysiology of liver and kidney diseases, various inflammation, skin diseases, and neurodegeneration.

Several previous studies have investigated the involvement of TGs in renal disease^5,9,15–18^ and showed that TG2 appears to play an important predominant role in the disease. Indeed, a TG2 knockout mouse (TG2KO) model was used to demonstrate a 50% suppression of increased fibrillary collagen, macrophage infiltration, and active TGF-β1 after unilateral ureteral obstruction (UUO)^16^. However, treatment with a TG inhibitor almost completely reduced elevated ε(γ-glutamyl) lysine crosslinking, hydroxyproline content, fibrotic area, and TGF-β1 activation after 5/6-nephrectomy in rat^15^. Although these results were evaluated in different animal and experimental model for fibrosis induction, TG inhibition rather than its genomic deficiency suggested an effective action for the prevention of fibrosis. These findings indicate that isozymes other than TG2 might have possible functions in fibrosis induction. Recently, Zhang *et al*. and Ponnusamy *et al*. reported that TG1 was highly expressed and activated by H_2_O_2_, oxidant injury, in a renal tubular epithelial cell line, whereas TG2 was not detectable^6,7^. Therefore, further detailed studies on the distributions of TG expression and activities as well as on the identification of disease-specific substrates for each TG isozyme are required.

We previously characterized preferred Gln-donor substrate sequences with a unique reaction tendency for each TG isozyme using a random peptide library^19,20^. These peptide sequences appeared to act as substrates with high reactivity and isozyme specificity, even in the peptide form (12 amino acid residues). Accordingly, peptides can be successfully applied to detecting isozyme-specific activities by their incorporation into the Lys residues of substrate proteins as a form of labeled-peptides^21^. Previous studies have shown that TG1 is mainly involved in skin formation, contributing to the barrier function of the outermost layers via crosslinking of structural proteins in keratinocytes^12,22^, whereas TG2 is widely distributed and plays multiple roles, including in apoptosis, signal transduction, matrix stabilization, wound healing, and angiogenesis^10^. Recently, we suggested that TG1 is involved in the functional modification of intracellular protein, whereas TG2 predominantly contributes to the stabilization of extracellular proteins in liver fibrosis^23^.

To elucidate the detailed mechanism by which TG1 and TG2 contribute to kidney fibrotic diseases, here we detected their isozyme-specific activities and performed comprehensive analyses by identifying the possible substrate proteins incorporated the peptides for TG1 and TG2, as well as biotinylated pentylamine (BPA). We also evaluated the enhanced activity of each TG isozyme, and globally identified the Lys-donor substrates for each TG isozyme and the Gln-donor substrates for the entire TG family during the induction of renal fibrosis. This showed that the modification of these possible substrate proteins via crosslinking by TGs might be involved in renal fibrosis. These results provide novel insights into the mechanisms of tissue fibrosis, identify possible targets for antifibrotic therapy, and will also be helpful in elucidating the physiological and pathological functions of TGs in an isozyme-dependent manner.

## Results

### Evaluation of fibrotic markers in renal fibrosis induced by UUO surgery

**Figure 1:**
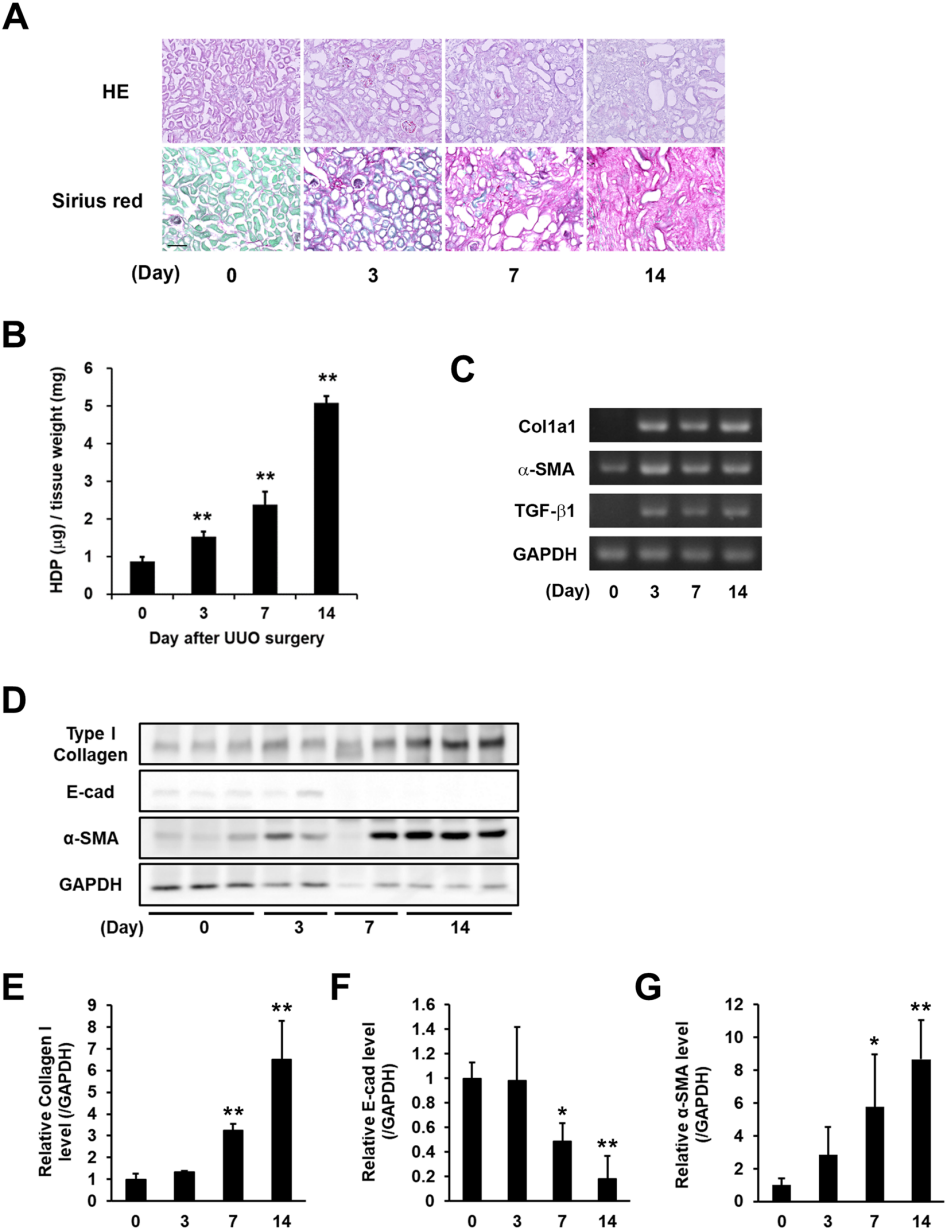
Evaluation of the fibrotic markers in kidney fibrosis after UUO surgery. UUO was induced in mice by ligating the left ureter in 8 weeks-old ICR mice. The mice were then sacrificed on days 3, 7, and 14 after surgery (*n* = 7–10 mice per group). (**A**) Kidney sections were fixed in 4% paraformaldehyde, and then stained using H&E and Sirius Red (using the Sirius Red Collagen Detection Kit). The red and green colors indicate the fibrillar collagen (type I to V collagen) and non-collagenous protein, respectively. Bar = 100 μm. (**B**) Hydroxyproline (HDP) contents were evaluated in the kidney on each indicated day after UUO surgery. The data are presented as the mean ± SD (*n* = 3) (**P < 0.01, Student’s *t*-test). (**C**) The mRNA expression levels of the fibrotic markers (Collagen Iα1 (Col1a1), α-SMA, TGF-β1, and GAPDH) were confirmed by RT-PCR. The full-length gel is presented in Fig. S1. (**D**) The protein levels in the whole lysate from the kidney tissue were analyzed by immunoblotting using each indicated antibody and GAPDH as a loading control for each sample. The full-length gel is presented in Fig. S2. (**E**–**G**) Relative changes in the densitometric profiles of collagen I, E-cadherin (E-cad), and α-SMA from (**D**) are presented under corresponding bands after normalizing to the changes in GAPDH. The data are presented as the mean ± SD (*n* = 4–5) (**P < 0.01, *P < 0.05, Student’s *t*-test).

Renal fibrosis was induced in mice by UUO surgery. To evaluate fibrotic levels, the accumulation of collagen was measured in the kidney at 3, 7, and 14 days after UUO surgery. H&E staining clearly indicated the renal tubular damage, which is characterized by dilation, flattening of epithelium, and expansion of interstitial areas resulting from UUO surgery. Sirius Red/Fast Green staining allowed collagen fibers, which appeared as well-defined, red-stained fibrillary elements compared to green-stained non-collagen components, to be clearly visualized. Sirius Red staining showed a dramatic increase in collagen around the renal tubular epithelial and interstitial areas at 3, 7, and 14 days (Fig. 1A). The hydroxyprolin (HDP) levels increased 1.7-fold at 3 days, 2.7-fold at 7 days, and 5.8-fold at 14 days compared with the control (Day 0; Fig. 1B), and levels of mRNA expression of fibrotic markers (collagen Iα1, α-SMA, and TGF-β1), analyzed by RT-PCR, were markedly higher in UUO-treated mice (Fig. 1C). In addition, the protein levels of collagen I, E-cadherin (E-cad; epithelial cell marker), and α-SMA were also presented in UUO-treated mice (Fig. 1D) with these relative values normalized to the changes in GAPDH (Fig. 1E–G), indicating the significant increase of fibrotic markers and decrease of epithelial-mesenchymal transition (EMT) marker.

### Measurement of the expression of TG isozymes during renal fibrosis

**Figure 2:**
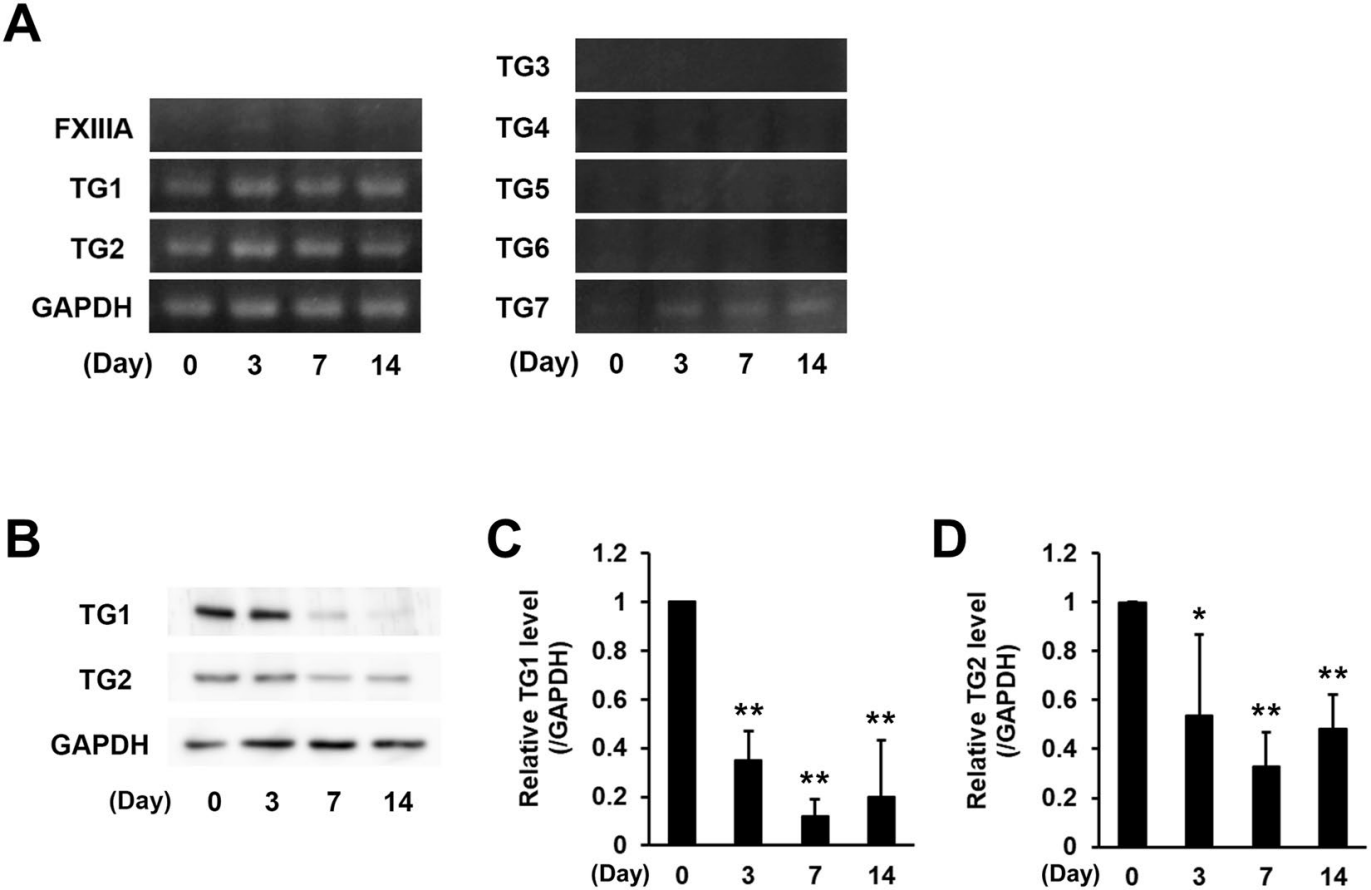
Expression levels of mRNA and protein of TG family in kidney fibrosis. (**A**) The mRNA expression levels of the TG family (FXIIIA and TG1–7) were confirmed by RT-PCR. The successful detections for TG3–6 using each primer pair are confirmed in the other tissue extracts. The full-length gels are presented in Fig. S3. (**B**) The protein levels in the whole lysate from the kidney tissue were analyzed by immunoblotting using each indicated antibody and GAPDH as a loading control for each sample. The full-length blot is presented in Fig. S4. (**C,D**) Relative changes in the densitometric profiles of TG1 and TG2 levels from (**B**) are presented under corresponding bands after normalizing to the changes in GAPDH. The data are presented as the mean ± SD (*n* = 3) (**P < 0.01, *P < 0.05, Student’s *t*-test).

To investigate the expression levels of TG family members, we confirmed the mRNA values of all TG isozymes (Fig. 2A). We found that mRNA levels of TG1 and TG2 were predominant in kidney, while mRNAs of the other TG isozymes, with the exception of TG7, were not detectable following a 40-cycle PCR analysis. Interestingly, the mRNA expression of TG7 increased marginally during renal fibrosis. The protein levels of TG1 and TG2 were also observed in UUO-treated mice (Fig. 2B). After UUO surgery, both TG1 and TG2 protein levels in the soluble fraction containing 0.1% TritonX-100 were found to be markedly decreased (Fig. 2B–D).

### Expression and activity distributions of TG1 and TG2 in fibrotic kidney

**Figure 3:**
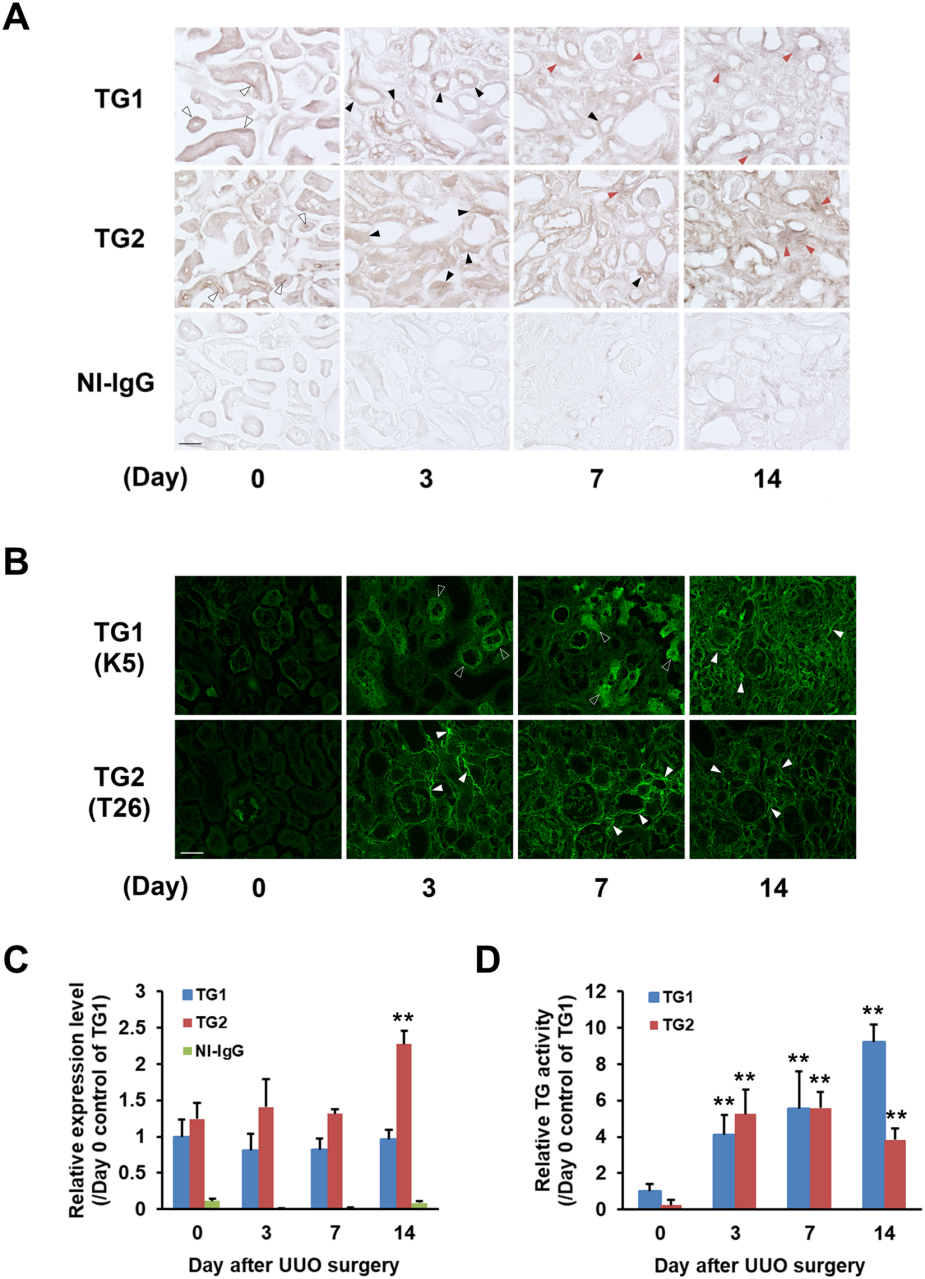
Distributions of the expressions and activities of TG1 and TG2 in fibrotic kidneys. Each kidney section was subjected to the immunohistochemistry and *in situ* TG activity staining on the indicated days after UUO surgery. (**A**) Immunostaining was performed using polyclonal anti-mouse TG1 and TG2 antibodies, and rabbit NI-IgG as the negative control. Three kinds of arrowheads indicate the areas where staining is changing for TG1 and TG2 such as tubule basement membrane and lumen (white arrowheads), tubular epithelial cells (black arrowheads), and interstitial areas (red arrowheads). Bar = 50 μm. (**B**) The *in situ* activities of TG1 and TG2 were visualized using FITC-labeled substrate peptides (pepK5 and pepT26, respectively). The white frame and white arrowheads indicated the notable tubular epithelial cells and interstitial areas, respectively. Bar = 50 μm. The relative intensities from (**A**) and (**B**) were presented in (**C**) and (**D**), respectively. The data are presented as the mean ± SD (*n* = 4) (**P < 0.01, Student’s *t*-test).

To evaluate the distributions of TG1 and TG2 expression, we immunostained kidney sections obtained at set days after UUO (Fig. 3A). The immunostaining data failed to show any reduced expression of TG1 and TG2 as previously shown by western blot analysis (Fig. 2B) in fibrotic kidney. Both TG1 and TG2 expression levels were increased in the interstitial area after UUO. The distribution of TG1 and TG2 appeared to be drastically altered in the renal tubule at day 3 after UUO compared with control kidney, where TG1 and TG2 expression was predominantly distributed around the tubule basement membrane and lumen, respectively.

We then visualized the *in situ* enzymatic activities of TG1 and TG2. In our system, isozyme-specific activities of TG1 and TG2 were measured using the substrate peptides pepK5 and pepT26, respectively^19,20^. This indicated that TG1 activity was enhanced in the renal tubule at the early fibrotic stage (Day 3) and in the extracellular space at late fibrotic stages, while TG2 activity was significantly enhanced in the interstitial area of the fibrotic kidney (Fig. 3B). No fluorescent signals were observed in reactions using their mutant forms of pepK5 and pepT26, in which Gln are replaced with asparagine (*data not shown*). The relative values obtained with these immunostaining and *in situ* activity staining indicated that the enhancement of TG1 and TG2 activities were more sensitive to the response in UUO surgery than those of their proteins (Fig. 3C,D).

**Figure 4:**
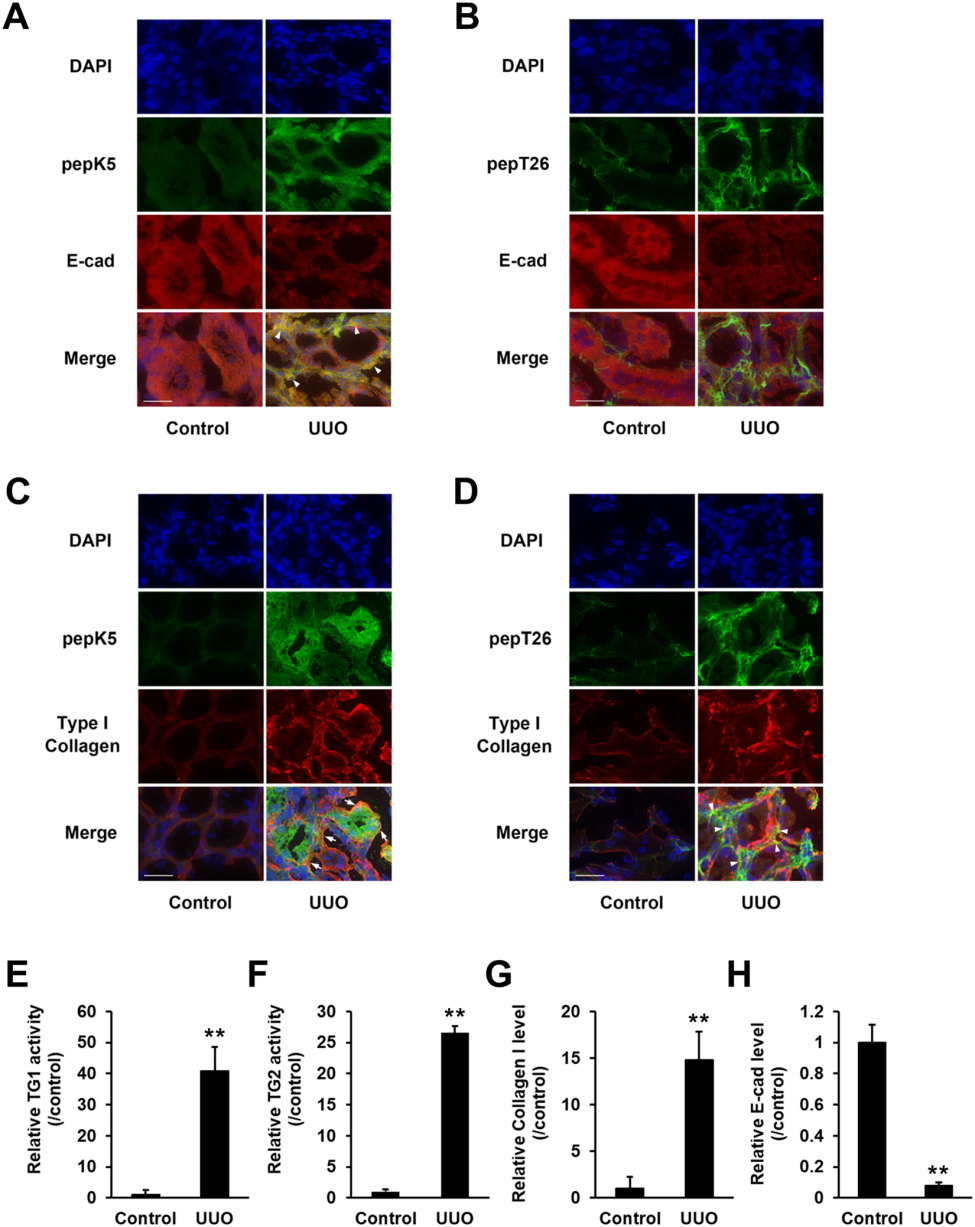
Detailed distribution analysis of the enhanced activities of TG1 and TG2. The colocalization of the activity of each TG with the E-cadherin or type I collagen in the kidney sections was analyzed in the control and at 3 days after UUO surgery. (**A,B**) The kidney sections were incubated with FITC-labeled substrate peptides. Following fixation in 4% paraformaldehyde, the sections were immunostained using anti-E-cadherin antibody and counterstained with DAPI. Merged staining images are shown in the bottom lane, with arrowhead indicating the similar distributions between E-cadherin and each TG. Bars = 25 μm. (**C,D**) The kidney sections were incubated with FITC-labeled substrate peptides. Following fixation in 4% paraformaldehyde, the sections were immunostained using anti-collagen type 1A1 antibody and counterstained using DAPI. Merged staining images are shown in the bottom lane, with arrow indicating the different distributions between collagen and each TG. Bars = 25 μm. (E-H) The relative fluorescence intensities from (**A–D**) were presented. The data are presented as the mean ± SD (*n* = 6–8) (**P < 0.01, Student’s *t*-test).

To further evaluate the distribution of each TG activity in detail, we analyzed their colocalization with E-cadherin and collagen (Fig. 4). Compared with control mice, the expression levels of E-cadherin dramatically decreased in renal tubular epithelium (Fig. 4A,B and H), while that of collagen increased around tubule basement membrane in UUO-treated mice (Fig. 4C,D and G). The enhanced activity of TG1 colocalized to the renal tubular epithelium where expression of E-cadherin was low (Fig. 4A,E and H), whereas TG2 activity partly overlapped with the localization of collagen I (Fig. 4D,F and G).

### Effect of TGs inhibitor or genetic defect of TG2 during renal fibrosis

**Figure 5:**
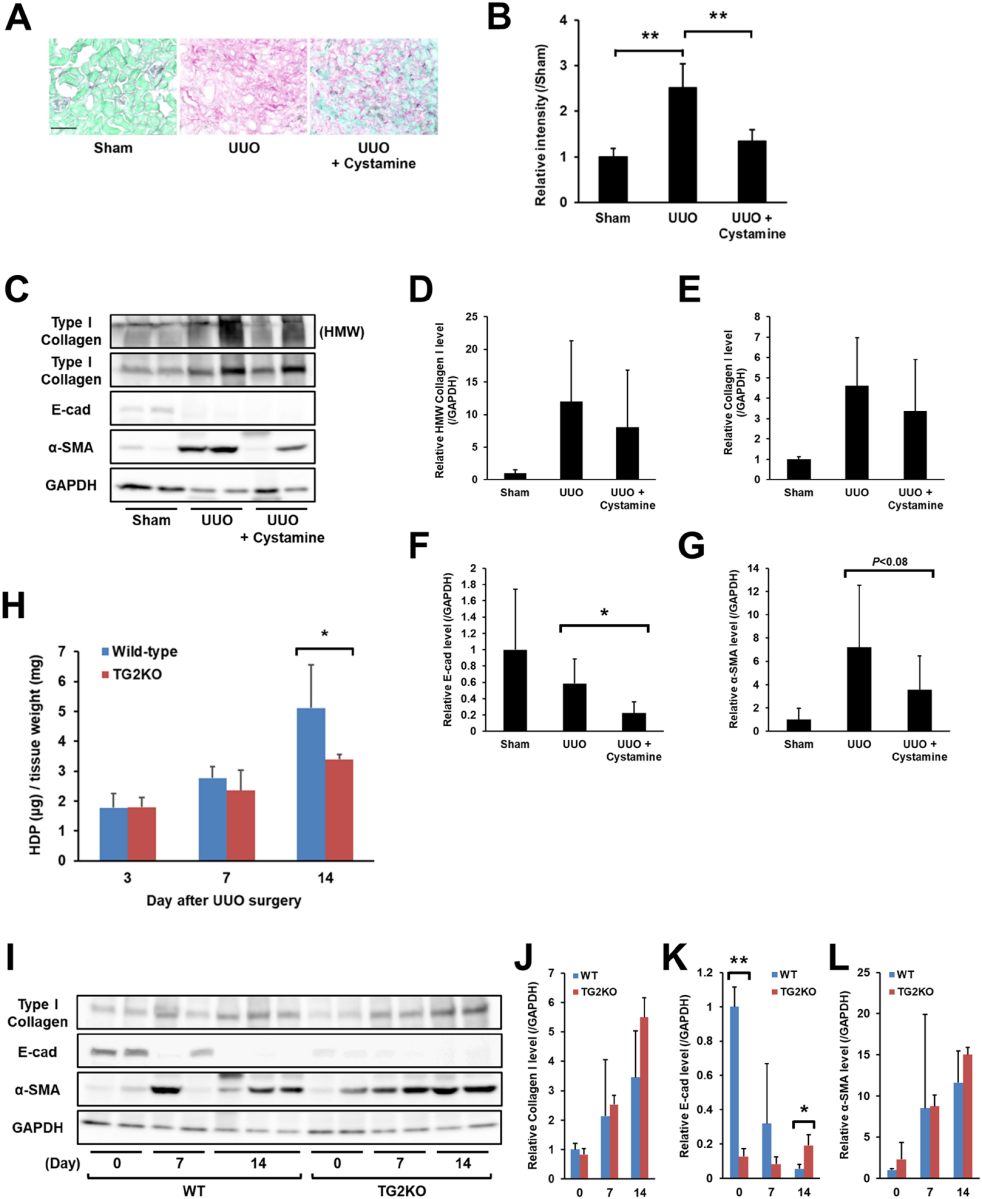
The effect of TGs inhibitor or genetic defect of TG2 during renal fibrosis. Cystamine, a competitive inhibitor for the crosslinking activity of TGs, was orally administrated in drinking water (approximately 8.75 mg/kg/day) from 2 days before the UUO surgery (*n* = 9–12 mice per group). At 14 days after UUO, the fibrotic kidney was collected and evaluated for collagen deposition. (**A**) The kidney sections were stained using a Sirius Red Collagen Detection Kit. The red and green colors indicate the fibrillar collagen (type I to V collagen) and non-collagenous protein, respectively. Bar = 100 μm. (**B**) The relative intensity of contents was also evaluated in the same kidney tissues. The data are presented as the mean ± SD (*n* = 4) (**P < 0.01, Student’s *t*-test). (**C**) The protein levels in the whole lysate of kidney tissue from cystamine-administrated mice were analyzed by immunoblotting using each indicated antibody and GAPDH as a loading control for each sample. The bands of collagen I in the position of high molecular weight (HMW) were separately presented in this figure. The full-length gel is presented in Fig. S6. (**D–G**) Relative changes in the densitometric profiles of collagen I, E-cadherin, and α-SMA from (**C**) are presented under corresponding bands after normalizing to the changes in GAPDH. The data are presented as the mean ± SD (*n* = 6–7) (*P < 0.05, Student’s *t*-test). (**H**) The wild-type and TG2KO mice were sacrificed on days 3, 7, and 14 after UUO surgery (*n* = 6–8 mice per group). The HDP contents were evaluated in the kidney on each indicated day after UUO surgery. The data are presented as the mean ± SD (*n* = 3) (*P < 0.05, Student’s *t*-test). (**I**) The protein levels in the whole lysate of kidney tissue from the WT and TG2KO mice after UUO surgery were analyzed by immunoblotting using each indicated antibody and GAPDH as a loading control for each sample. The full-length gel is presented in Fig. S7. (**J**–**L**) Relative changes in the densitometric profiles from (**D**) are presented under corresponding bands after normalizing to the changes in GAPDH. The data are presented as the mean ± SD (*n* = 3–4) (**P < 0.01, *P < 0.05, Student’s *t*-test).

To confirm whether the crosslinking activities of TGs promote fibrosis in the kidney, cystamine, a competitive inhibitor of TG activity, was orally administered to UUO-treated mice (Fig. 5). As anticipated, cystamine treatment reduced collagen deposition at 14 days after UUO accompanied with the reduced activities of TG1 and TG2 (Figs 5A and S5). The values obtained with Sirius Red staining indicated that cystamine treatment decreased the enhanced relative intensity in UUO-treated mice by 80% (Fig. 5B). In the analyses of protein levels by western blot, the levels of soluble collagen I tended to decrease in cystamine treatment at same days after UUO (Fig. 5C–E), and the levels of E-cadherin and α-SMA were considerably reduced, though the difference of α-SMA level were not statistically significant (Fig. 5C,F, and G). Additionally, levels of HDP elevated after UUO were reduced by approximately 50% in TG2KO mice compared to wild-type mice (Fig. 5H), which is consistent with the results of Shweke *et al*.^16^, although the soluble protein levels of collagen I and α-SMA showed no alteration (Fig. 5I,J and L). Interestingly, TG2KO mice showed the significant difference in the level of E-cadherin at control and 14 days after UUO (Fig. 5I,K). These findings suggest that isozymes other than TG2 might also have a possible function in renal fibrosis induction.

### Effect of TGs inhibitor or knockdown of TG1 and TG2 in renal tubular epithelial cells

**Figure 6:**
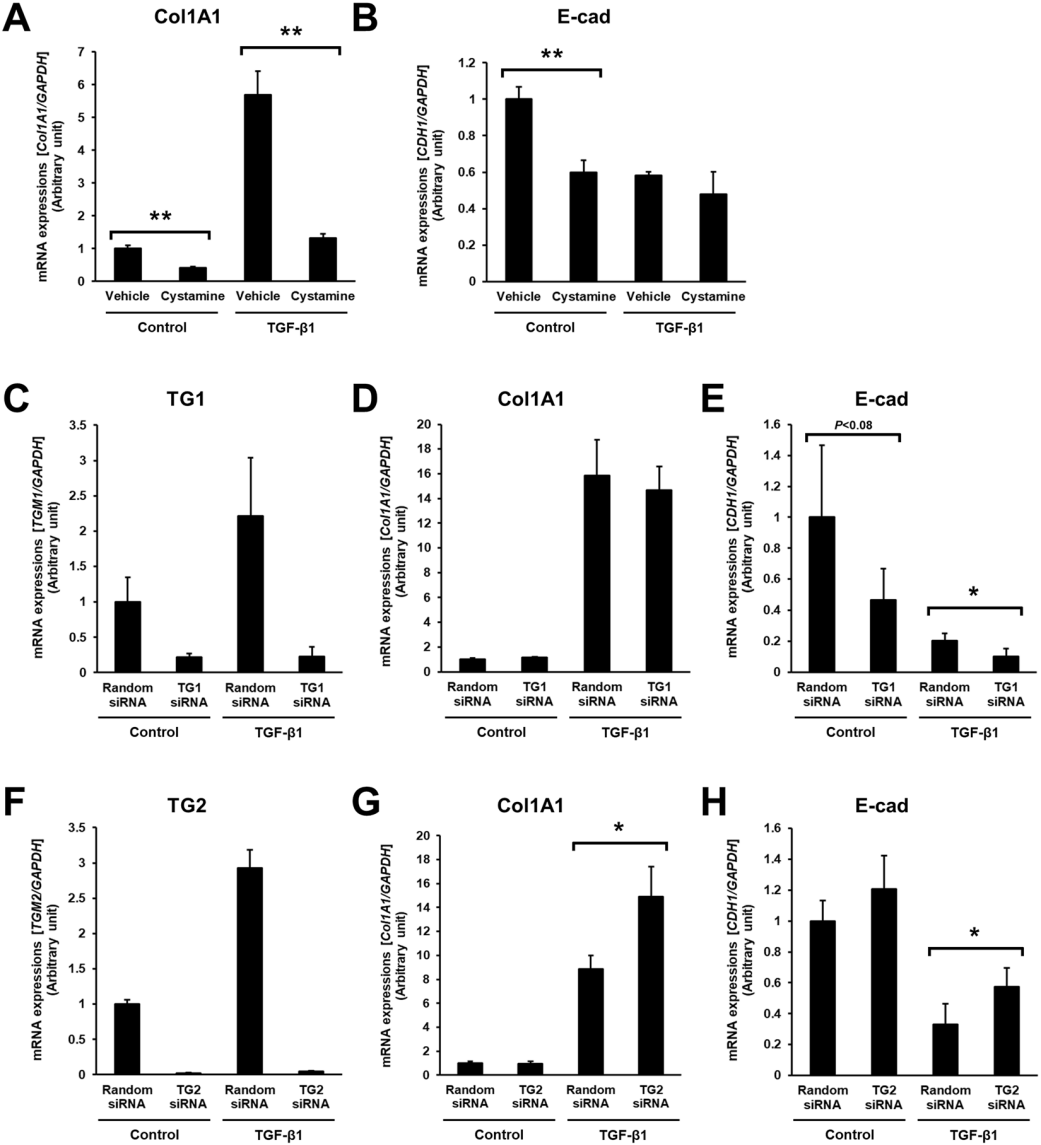
The effect of TGs inhibitor or knockdown of TG1 and TG2 in renal tubular epithelial cells. (**A,B**) Human tubular epithelial cells (HK-2) were plated in 6 cm dish (3 × 10^5^ cells) and treated with 5 ng/ml TGF-β1 in the presence or absence of 1 mM cystamine in serum-free medium for 12 h. (**C–H**) For the knockdown of specific gene, siRNA mixture against TG1 (**C–E**) or TG2 (**F–H**) were added on the culture dish before seeding the cells. As a negative control, scrambled siRNA (Random) was replaced with the same amount of siRNA against TG1 and TG2. Then, after 24 h incubation, cells were treated with TGF-β1 for 12 h. Total mRNA were isolated and the indicated mRNA levels were evaluated using quantitative RT-PCR. Data were normalized against GAPDH mRNA expression and relative value (a ratio of the control sample) were presented as the mean ± SD (*n* = 3) (**P < 0.01, *P < 0.05, Student’s *t*-test).

To further observation in the role of TG activity in tubular epithelium during renal fibrosis, the *in vitro* model of tubular epithelial cells (HK-2) with treatment of TGF-β1 as an inducer of ECM and EMT was used in the presence of TG inhibitor or siRNA of TG1 and TG2. The cystamine treatment in the HK-2 completely suppressed the mRNA level of collagen Iα1 enhanced by TGF-β1 although the mRNA level of E-cadherin was also reduced (Fig. 6A,B). However, the knockdown of TG1 and TG2 by their siRNAs failed to show the reduction of collagen Iα1 mRNA (Fig. 6D,G), while the mRNA level of E-cadherin was decreased with the knockdown of TG1 and was increased with that of TG2 (Fig. 6E,H). These findings suggest that TG1 and TG2 might involve in the EMT but not the production of collagen Iα1 during renal fibrosis induction.

### Comprehensive identification of possible substrates in fibrosis promotion

**Figure 7:**
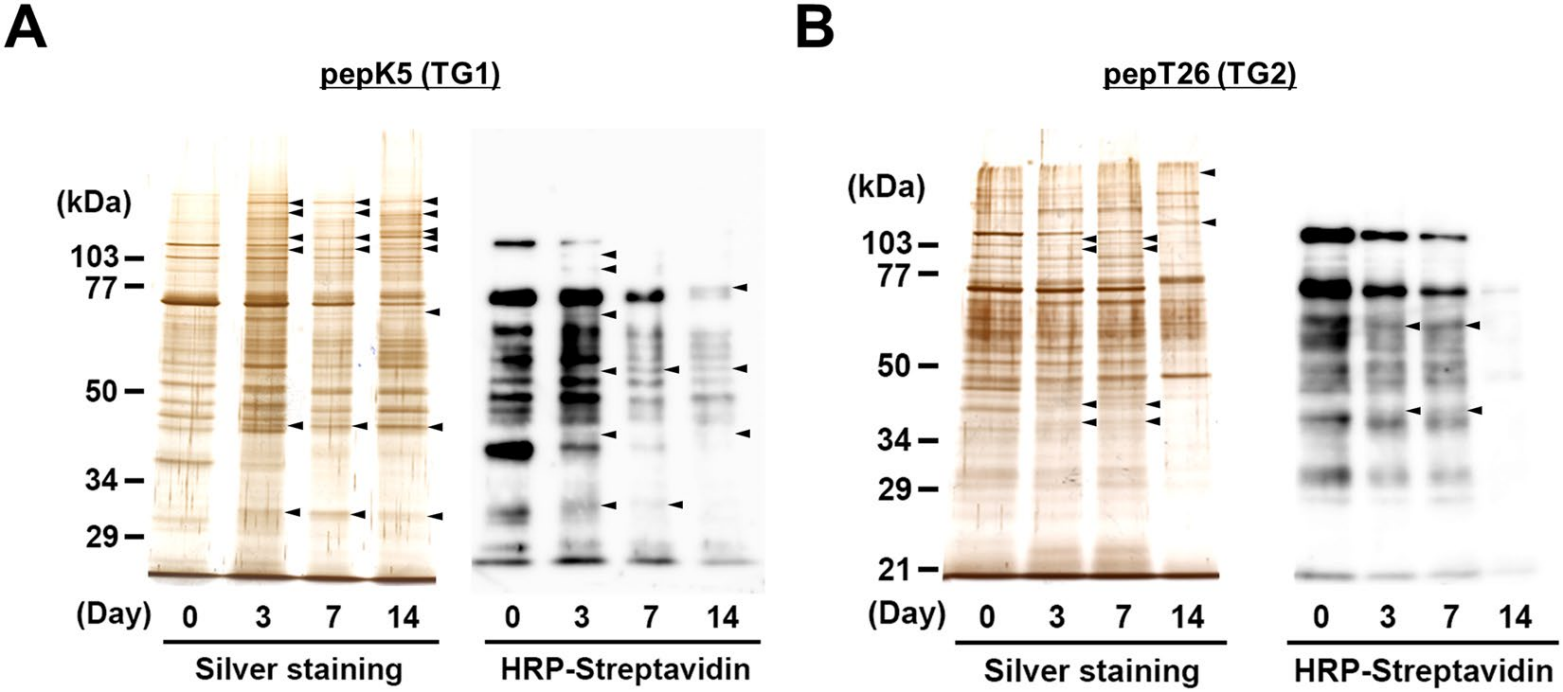
Detection of possible substrates incorporated with each peptide in kidney extracts. Each kidney extract on the indicated days after UUO surgery was incubated with the biotinylated pepK5 (for TG1; **A**) and pepT26 (for TG2; **B**). These biotinylated peptide-incorporated proteins after the purification using monoavidin gel were subjected to silver staining or detection using peroxidase-conjugated streptavidin. The sizes of the protein mass markers are shown on the left. Arrowheads indicate the bands that increased compared with the control sample (Day 0). Each experiment was done with more than three replicates from at least three independent experiments.

To further investigate TG activities during renal fibrosis, we detected Lys- and Gln-donor substrates crosslinked by TGs. Each biotinylated substrate peptide or BPA were incubated with kidney extracts obtained at set days after UUO surgery; the resultant proteins that incorporated the peptide or BPA were purified using monoavidin gel and then detected by silver staining or development using peroxidase-conjugated streptavidin after SDS-PAGE (Figs 7A,B and S8). These purified proteins were also subjected to trypsin digestion for identification using MALDI-TOF/TOF mass spectrometer. This resulted in the identification of 47 and 67 possible substrates for TG1 and TG2, respectively, all of which were identified only in fibrotic kidney and not in untreated control (Tables 1,2, S1,S2). By similar procedures, a total of 67 unique proteins incorporating BPA were also identified as TG substrates (Tables S3 and S4). Interestingly, the fibrotic markers collagen and α-SMA were included among the possible substrates identified for TG1 but not for TG2.

**Table 1:** Identified possible substrates for TG1 using pepK5.

Accession number	Name	Days			
		0	3	7	14
P01029	Complement C4-B		+	+	+
Q8K0E8	Fibrinogen β chain		+	+	+
Q922U2	Keratin, type II cytoskeletal 5		+	+	+
P31725	Protein S100-A9		+	+	+
Q921I1	Serotransferrin		+	+	+
P68373	Tubulin α-1C chain		+	+	+
P99024	Tubulin β-5 chain		+	+	+
P62270	40 S ribosomal protein S18		+	+	
Q8R1M2	Histone H2A.J		+	+	
Q68FD5	Clathrin heavy chain 1		+		+
O35737	Heterogeneous nuclear ribonucleoprotein H		+		+
P62737	Actin, aortic smooth muscle			+	+
P06909	Complement factor H			+	+
Q8VCM7	Fibrinogen γ chain			+	+
Q9Z2X1	Heterogeneous nuclear ribonucleoprotein F			+	+
Q91X17	Uromodulin			+	+
Q8CGP1	Histone H2B type 1-K		+		
P62960	Nuclease-sensitive element-binding protein 1		+		
Q9WV32	Actin-related protein 2/3 complex subunit 1B			+	
Q8BSL7	ADP-ribosylation factor 2			+	
Q8JZQ5	Amiloride-sensitive amine oxidase [copper-containing]			+	
P10126	Elongation factor 1-α 1			+	
P16110	Galectin-3			+	
P11499	Heat shock protein HSP 90-β			+	
P02104	Hemoglobin subunit ε-Y2			+	
Q99020	Heterogeneous nuclear ribonucleoprotein A/B			+	
Q8VEK3	Heterogeneous nuclear ribonucleoprotein U			+	
Q8CGP7	Histone H2A type 1-K			+	
P10854	Histone H2B type 1-M			+	
O89017	Legumain			+	
P26041	Moesin			+	
P17225	Polypyrimidine tract-binding protein 1			+	
Q61656	Probable ATP-dependent RNA helicase DDX5			+	
P26043	Radixin			+	
Q62093	Splicing factor, arginine/serine-rich 2			+	
Q78PY7	Staphylococcal nuclease domain-containing protein 1			+	
P26039	Talin-1			+	
Q9CWF2	Tubulin β-2B chain			+	
P60710	Actin, cytoplasmic 1				+
P63268	Actin, γ-enteric smooth muscle				+
Q60847	Collagen α-1(XII) chain				+
P01942	Hemoglobin subunit α				+
P02089	Hemoglobin subunit β-2				+
P01873	Ig mu chain C region membrane-bound form				+
P08071	Lactotransferrin				+
P11247	Myeloperoxidase				+
P68369	Tubulin α-1A chain				+

Kidney extract on each indicated day after UUO surgery was incubated with biotinylated pepK5. The newly identified possible substrates in each indicated day were demonstrated as “+” compared to control sample (Day 0). The underlined possible substrates indicate overlapped substrates identified as both peptide (pepK5 and pepT26)-incorporated proteins. This Each experiment was done with two replicates from three independent experiments.

**Table 2:** Identified possible substrates for TG2 using pepT26.

Accession number	Name	Days			
		0	3	7	14
O08756	3-hydroxyacyl-CoA dehydrogenase type-2		+	+	+
P20060	β-hexosaminidase subunit β		+	+	+
P05064	Fructose-bisphosphate aldolase A		+	+	+
Q91Y97	Fructose-bisphosphate aldolase B		+	+	+
P54071	Isocitrate dehydrogenase [NADP], mitochondrial		+	+	+
P51660	Peroxisomal multifunctional enzyme type 2		+	+	+
P31725	Protein S100-A9		+	+	+
P26040	Ezrin		+	+	
P06909	Complement factor H		+		+
P63017	Heat shock cognate 71 kDa protein		+		+
O35737	Heterogeneous nuclear ribonucleoprotein H		+		+
Q61781	Keratin, type I cytoskeletal 14		+		+
P08071	Lactotransferrin		+		+
P45952	Medium-chain specific acyl-CoA dehydrogenase, mitochondrial		+		+
P26041	Moesin		+		+
Q99MZ7	Peroxisomal trans-2-enoyl-CoA reductase		+		+
Q9WUA2	Phenylalanyl-tRNA synthetase β chain		+		+
P84104	Splicing factor, arginine/serine-rich 3		+		+
P26443	Glutamate dehydrogenase 1, mitochondrial			+	+
Q9WVE8	Protein kinase C and casein kinase substrate in neurons protein 2			+	+
P68373	Tubulin α-1C chain			+	+
P99024	Tubulin β-5 chain			+	+
Q60597	2-oxoglutarate dehydrogenase, mitochondrial		+		
Q9D8E6	60 S ribosomal protein L4		+		
P62897	Cytochrome c, somatic		+		
Q99LB2	Dehydrogenase/reductase SDR family member 4		+		
P58252	Elongation factor 2		+		
Q64525	Histone H2B type 2-B		+		
Q9ERE2	Keratin, type II cuticular Hb1 (Fragment)		+		
Q78PY7	Staphylococcal nuclease domain-containing protein 1		+		
Q9ERD7	Tubulin β-3 chain		+		
P62270	40 S ribosomal protein S18			+	
Q6ZWV3	60 S ribosomal protein L10			+	
P60710	Actin, cytoplasmic 1			+	
P48962	ADP/ATP translocase 1			+	
Q03265	ATP synthase subunit α, mitochondrial			+	
P56480	ATP synthase subunit β, mitochondrial			+	
Q8BFZ3	β-actin-like protein 2			+	
P16858	Glyceraldehyde-3-phosphate dehydrogenase			+	
P30681	High mobility group protein B2			+	
Q9D646	Keratin, type I cuticular Ha4			+	
Q6IMF0	Keratin, type II cuticular Hb3			+	
Q8R429	Sarcoplasmic/endoplasmic reticulum calcium ATPase 1			+	
P05214	Tubulin α-3 chain			+	
P68372	Tubulin β-2C chain			+	
P28653	Biglycan				+
Q9CZU6	Citrate synthase, mitochondrial				+
Q9QZQ8	Core histone macro-H2A.1				+
Q99020	Heterogeneous nuclear ribonucleoprotein A/B				+
Q9Z130	Heterogeneous nuclear ribonucleoprotein D-like				+
Q9Z2X1	Heterogeneous nuclear ribonucleoprotein F				+
Q8VEK3	Heterogeneous nuclear ribonucleoprotein U				+
P63158	High mobility group protein B1				+
Q8R1M2	Histone H2A.J				+
Q9D2U9	Histone H2B type 3-A				+
P84244	Histone H3.3				+
P62960	Nuclease-sensitive element-binding protein 1				+
P09405	Nucleolin				+
Q8BK67	Protein RCC2				+
P27005	Protein S100-A8				+
P54276	REVERSED DNA mismatch repair protein Msh6				+
Q921I1	Serotransferrin				+
P26039	Talin-1				+
Q9D0R2	Threonyl-tRNA synthetase, cytoplasmic				+
Q9CWF2	Tubulin beta-2B chain				+
Q91X17	Uromodulin				+
P50544	Very long-chain specific acyl-CoA dehydrogenase, mitochondrial				+

Kidney extract on each indicated day after UUO surgery was incubated with biotinylated pepT26. The newly identified possible substrates in each indicated day were demonstrated as “+” compared to control sample (Day 0). The underlined possible substrates indicate overlapped substrates identified as both peptide (pepK5 and pepT26)-incorporated proteins.

Seven proteins were reproducibly identified in all reaction mixtures for extracts obtained at set days after UUO (Day 3, 7, and 14), excepting the control (Day 0), as possible TG1 substrates (Complement C4-B, Fibrinogen β chain, Keratin 5, Protein S100-A9, Serotransferrin, Tubulin α-1C, and Tubulin β-5; Table 1) and TG2 substrates (3-hydroxyacyl-CoA dehydrogenase type-2, β-hexosaminidase subunit β, Fructose-bisphosphate aldolase A, Fructose-bisphosphate aldolase B, Isocitrate dehydrogenase, Peroxisomal multifunctional enzyme type 2, and Protein S100-A9; Table 2).

### Functional categorization of identified isozyme-specific TG substrates

**Figure 8:**
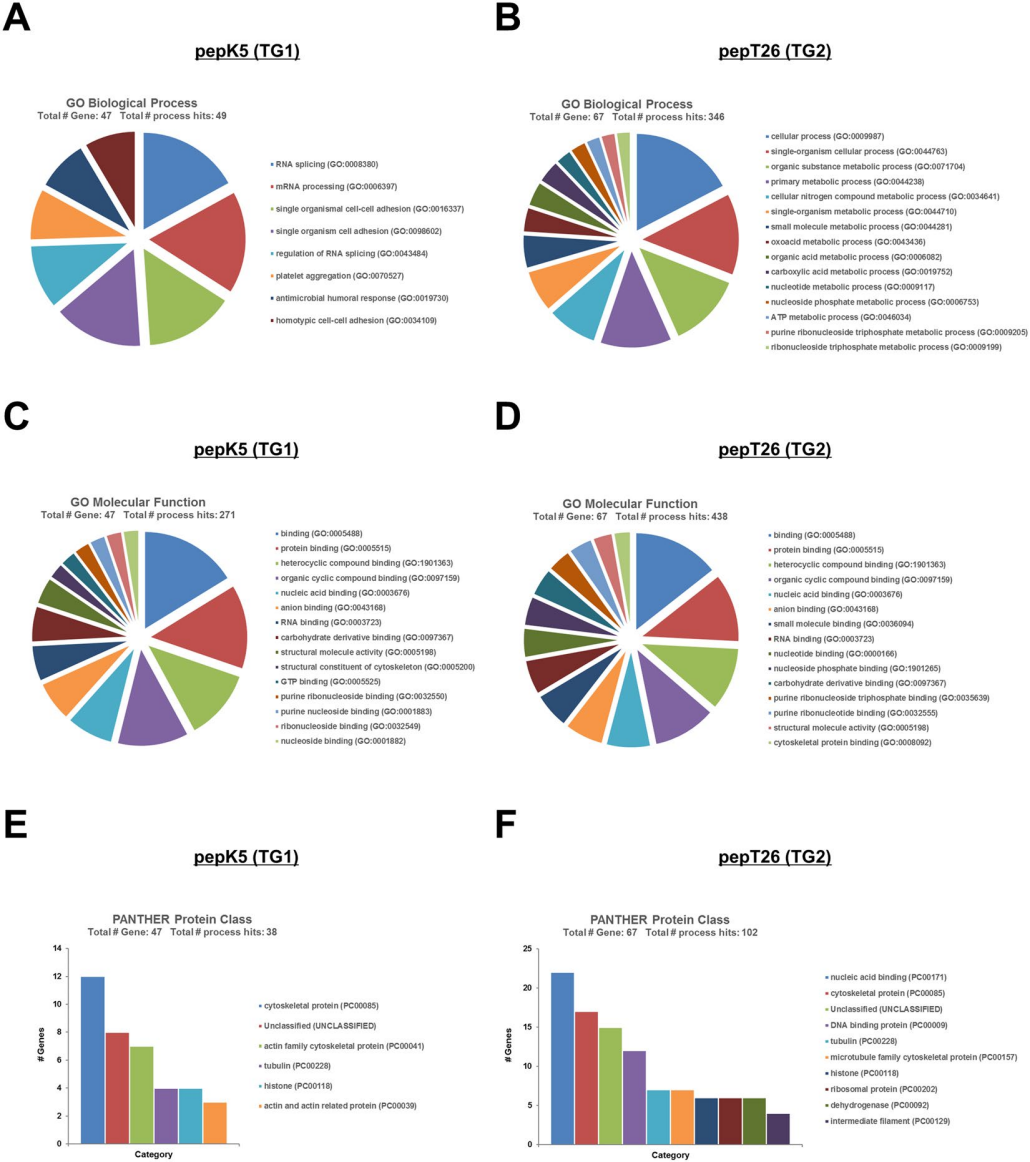
Gene ontology (GO) terms for possible substrates for TG1 and TG2 during renal fibrosis. GO analysis corresponding to biological process (**A**), molecular function (**B**) and PANTHER protein class (**C**) represented as pie and bar charts generated by PANTHER classification system (http://www.pantherdb.org/).

The identified proteins were annotated with Gene Ontology (GO) information from the Uniprot database using Gene Ontology Consortium (http://geneontology.org/) and Protein Analysis Through Evolutionary Relationships (http://pantherdb.org/). The results, describing no more than 15 of the identified genes, are shown in Figs 8 and S9. The identified substrates for TG1 were categorized into 8 biological processes, of which both RNA splicing and mRNA processing comprised the highest percentages of genes (17.0%) (Fig. 8A). The single organismal cell-cell adhesion (14.9%) was subcategorized into the single organism cell adhesion category (14.9%). TG2-specific substrates were classified into 33 biological processes (a maximum of 15 are shown in the pie charts); the categories cellular process (17.3%) and single organism cellular process (13.6%) comprised the highest percentages of genes (Fig. 8B). Subsequently, the categories organic substance and primary metabolic process included 12.4% and 11.8% of genes, respectively. The molecular function attributed to substrates identified for both TG1 and TG2 were similarly categorized into binding, protein binding, heterocyclic and organic cyclic compound binding, with high percentages of genes (16.2%, 14.0%, and 11.8% for TG1 substrates, and 14.4%, 11.4%, and 10.5% for TG2, respectively) (Fig. 8C,D). When categorized according to protein class, most TG1-specific substrates were categorized as cytoskeletal proteins (31.6%) and actin family cytoskeletal proteins (18.4%), while TG2-specific substrates included proteins categorized as nucleic acid binding (21.6%), cytoskeletal protein (16.7%), and DNA binding protein (11.8%) (Fig. 8E,F). Interestingly, the identified Gln-substrates that incorporated BPA were categorized into 66 biological processes, although with the exception of the cellular process category (11.1%), most with relatively higher percentages of genes were not significant for the categorization (Fig. S9A). In terms of molecular function, Gln-substrates were categorized into binding (14.4%), protein binding (11.4%), heterocyclic and organic cyclic compound binding (10.7%), with high percentages of genes (Fig. S9B). With regard to protein class, the largest categories were nucleic acid binding (21.3%), RNA binding protein (12.9%), and cytoskeletal protein (11.8%) (Fig. S9C). Interestingly, identified substrates for TG1 showed a significant relationship with the cellular process category due to RNA splicing and processing, and cell adhesion, whereas those for TG2 were related to several metabolic processes. These results suggest that the substrates identified using each peptide were not uniformly characterized, whereas the BPA-incorporated substrates for pan TGs were uniformly categorized, with relatively lower percentage of genes in each category.

## Discussion

All types of CKD that progress to kidney failure are induced via common pathway of kidney fibrosis and scarring. Therefore, understanding the mechanisms involved in these processes is essential for the development of antifibrotic therapies. The crosslinking enzyme transglutaminase has a well-established role in altering the extracellular homeostatic balance, which leads to the excessive accumulation of ECM in the kidney that underlies CKD^5,24,25^. TGs comprise a large family of eight closely related but distinct isozymes and are widely distributed across specific tissue types and cells, where they are involved in multiple biological processes. The three major TG isozymes, namely TG1, TG2, and FXIII, have been investigated with regard to their structures, tissue and cellular distributions, activities, substrates, and relationship with diseases.

Most previous *in vivo* studies on kidney diseases have limited their attention to analyzing only TG2^5,9,15–17,24,25^. However, our previous study demonstrated that the expression and activity of TG1 significantly increased in renal tubular epithelium in the early phase of acute kidney injury^18^. Moreover, the present study also showed a marked enhancement of TG1 activity from the early stage of renal fibrosis. In addition, *in vitro* studies demonstrated that TG1 activity, but not its expression, was highly enhanced in oxidant-treated renal tubular epithelial cells^6,7^.

In this study, renal fibrosis was induced by UUO, which is commonly used as a model of experimental kidney fibrosis, although a disadvantage of this mouse model is the lack of possibility to estimate kidney function from serum creatinine and blood urea nitrogen, because of the compensatory changes of another non-obstructed kidney^26,27^. The progression of renal fibrosis was characterized by dilation, flattening of epithelium, and expanded interstitial areas. In addition, the marked accumulation of collagen Iα1, α-SMA, and TGF-β1 was observed at 3, 7 and 14 days after UUO surgery. Among all the TG isozymes, mRNA expression of TG1 and TG2 was detectable at the same level and relatively higher compared to the other isozymes. Interestingly, TG7 mRNA expression was increased in fibrotic kidney, although its expression level was lower than those of TG1 and TG2. This expression profile of the TG family in mouse kidney was different to that in rat kidney, which showed high TG2 and TG3 expression levels but low TG1 and TG7 expression levels^5^.

To exhaustively assess the role of the TG family members, we investigated the expression and enzymatic activity distribution of each TG. Labeled primary amines, including BPA, have been used to measure TG activities by evaluating the amount incorporated into Gln-donor substrates^28–30^. However, this does not allow the TG isozyme-specific activity to be measured. Therefore, we took advantage of specific substrate peptides for TG1 and TG2 to evaluate each TG activity in an isozyme-specific manner during the induction of renal fibrosis. Our results showed that the activities of TG1 and TG2 significantly increased in UUO-treated kidney (Fig. 3B). In the early stage of renal fibrosis (Day 3), *in situ* TG1 activity was markedly enhanced in the renal tubule, whereas TG2 activity increased only in the interstitial area; in later stages of renal fibrosis (Day 7 and 14), TG1 activity was strongly distributed in both the renal tubule and interstitial area, whereas TG2 activity localized to the extracellular space, including the area around the renal tubule and interstitial area. No elevated TG1 and TG2 activities were observed in glomeruli, as UUO is a specific model for renal interstitial fibrosis; thus, glomerular fibrosis was not prominent^31^. The enhanced TG1 activity in the renal tubule was confirmed in the colocalization with the renal epithelial marker, E-cadherin (Fig. 4A). Interestingly, the distribution of TG1 activity showed the opposite correlation with that of E-cadherin after UUO surgery, suggesting that TG1 activity might involve the EMT of renal epithelium following UUO surgery. In contrast, TG2 activity was observed to be enhanced around the renal tubule and interstitial area, with a distribution partly overlapping that of collagen I (Fig. 4D). In these data, the fluorescence intensities of E-cadherin and collagen in the areas in which they colocalize with the enhanced TG activity appear to be moderated. This might be due to a decrease in the antigenicity of E-cadherin and collagen caused by structural modification brought about by crosslinking. Furthermore, the analyses of proteins including crosslinked insoluble collagen such as HDP content and Sirius red staining demonstrated the marked reduction of collagen deposition in both cystamine-treated and TG2 knockout mice compared to these corresponding controls (Fig. 5A,B and H), although the western blot analysis showed little change levels of collagen I (Fig. 5D,E and J). In addition, the alteration of biological parameters including body weight, blood glucose, and kidney weight were not statistically significant in cystamine treatment and knockout of TG2 compared to these corresponding controls (Tables S5 and S6). Because it was well-known that TG2 contributes to collagen stabilization via crosslinking in the fibrosis induction, the western blot analysis for detecting mainly soluble collagen might not be enough to evaluate the amount of ECMs. The cystamine treatments in both UUO-treated mice and renal tubule epithelial cells demonstrate the 50% and 40% suppressions of E-cadherin, respectively (Figs 5F and 6B). The similar reduction of E-cadherin was observed in TG1 siRNA-treated cells as shown in Fig. 6E. These findings suggest that TG1 plays a potential role in the modification of intracellular substrates via crosslinking in the renal tubule epithelium, whereas extracellular TG2 contributes to the stabilization and maturation of fibrous proteins such as collagen and fibronectin^5,32^.

Immunostaining analysis showed a partial correlation in the distribution between the enhanced activity of TG1 and its expression. The increased expressions of both TG1 and TG2 were observed only in the interstitial area but not in other areas, including the renal tubule and glomerulus (Fig. 3A). Our previous study and others also demonstrated that the enzymatic activity of TGs did not completely correlate with their expression levels^7,23,33,34^. These results suggest that TG1, which is anchored to the inner surface of the plasma membrane in keratinocytes^35^, may exert its crosslinking activity in the renal tubule through activation mechanisms such as binding of activator and limited proteolysis^33,36^ during the induction of renal fibrosis. Western blot analysis showed that the expression levels of both TG1 and TG2 were marginally decreased at 7 and 14 days after UUO surgery, although mRNA expression analysis, immunostaining, and activity staining did not reflect these reduced expressions (Figs 2 and 3). These discrepancies might be attributable to a reduced antigenicity against its antibody, suggesting that TGs could become favorable substrates through crosslinking activity, which would result in polymerization, and could also possibly become susceptible to other modifications such as sumoylation and phosphorylation^37,38^.

The TG family is generally considered to exhibit high substrate specificity. Even for the same substrate protein, TG isozymes differ with regard to their reactivity and specificity^39^. Among their family, a number of articles reported that TG2 predominantly appears to have substantial role in renal disease^5,9,15–18^. Indeed, TG inhibitor NUT281 almost completely suppressed the induction of renal fibrosis in rat subjected to 5/6-nephrectomy^15^, whereas TG2KO mice showed a partial reduction in the progress of renal fibrosis in UUO-treated mice^16^. From these investigations, it would appear that the inhibitor of TG rather than the genomic deficiency of TG2 showed a higher protective effect against renal fibrosis. As NTU281 is non-selective for the TG family^40^, these results suggest that isozymes other than TG2 might also have a possible function for the induction of renal fibrosis. In addition, several articles indicated that TG1 was highly expressed and activated by oxidative stress in renal tubular epithelial cell line^6,7^, which is consistent with our results. As anticipated, reduced renal fibrosis accompanied by decreased collagen deposition was observed following treatment with the TG inhibitor cystamine and in TG2KO mice. These data also support our above hypothesis regarding the substantial role of TG1 in renal fibrosis, as cystamine treatment rather than TG2 deficiency showed a higher inhibitory effect on UUO-induced fibrosis (88% vs. 53%). Furthermore, we previously confirmed the involvement of other isozymes except TG2 in a mouse liver fibrosis model following bile duct ligation^23^. Cystamine treatment reduced liver fibrosis, whereas TG2KO mice showed similar fibrosis levels as wild-type mice. We hypothesized that this is causally related to compensation by other isozymes such as TG1^41^.

The identification of possible TG1/TG2 substrates using each substrate peptide supported the notion that both TG1 and TG2 are activated for crosslinking distinct substrates in an isozyme-independent manner in different areas of renal tissue. Of the possible TG1 and TG2 substrates identified here, only five (tubulin α-1C chain, 40S ribosomal protein S18, Histone H2A.J, staphylococcal nuclease domain-containing protein 1, and actin cytoplasmic 1) were found to be common substrates. However, we were unable to identify fibrotic marker proteins other than fibronectin, except for type XII collagen, which interacts with type I collagen-containing fibrils. In contrast, the extracellular fibrotic marker fibronectin was included only in the possible substrates identified using BPA. This is possibly due to the insolubility of these proteins, which is a result of excessive crosslinking; only soluble proteins can be crosslinked with each peptide and BPA and identified as a possible substrate by this method. In addition, the potential substrate proteins identified were limited to those substrates having unmodified Gln and Lys residues, as highly crosslinked substrates may already be crosslinked with endogenous substrates *in vivo*. Their Gln and Lys sites may, therefore, be occupied completely, thereby interfering in any new reaction with peptides and BPA. This hypothesis was reflected in the reduced number of possible substrates available to react with each peptide on Day 14 after UUO surgery. Moreover, the Gln sites in these substrates were limited, as Lys-donor peptides, substrates, and primary amines tend to have a lower specificity for each isozyme in the crosslinking reaction. Therefore, the exploration of the counterpart against identified possible substrates using Lys-donor substrates is an important next step.

The identification of substrates using biotinylated peptides for TG1 and TG2 in this study were performed as an initial trial and the results revealed several overlapping and abundant proteins. To develop a better understanding of the unique substrates for TG1 and TG2, further optimization regarding the purification and fractionation step of cellular components, including nucleus, membrane, and extracellular proteins, are underway in our laboratory.

We comprehensively identified various possible substrates using biotinylated substrate peptides for TG1 and TG2. However, we realize that the substrate peptide-incorporated proteins are not necessarily *in vivo* crosslinked proteins in renal fibrosis. So far, several researchers have used biotinylated primary amines such as BPA to measure TG activities and have identified possible substrates that serve as Gln donors^28–30^. We also believe that bait-molecules such as substrate peptides and primary amines enable the specific identification of possible substrates for each TG isozyme. Indeed, we previously demonstrated that most of the proteins which incorporate TG1-specific substrate peptides were well-known substrates crosslinked by TG1 with regards to the formation of envelope in osteoblast^42^ and cultured differentiated keratinocytes^43^. In this study, we identified several possible substrates, including ceruloplasmin^44^, heterogeneous nuclear ribonucleoprotein F^45^, legumain^46,47^, moesin^48^, myeloperoxidase^49^, protein S100-A9^50^, serotransferrin^51,52^, and uromodulin^53^, which are involved in the renal disease and fibrosis, although investigation into their relevance with TGs is ongoing. We speculate that the functional conversions of these substrates via crosslinking by TGs is involved in the progression of renal disease and fibrosis. Further investigations into the detailed mechanisms involved and biochemical analyses of these crosslinked substrates are also ongoing.

In conclusion, we determined the enhancement and distribution of each TG activity using isozyme-specific substrate peptides in fibrotic kidneys from UUO-treated mice. In addition, we comprehensively identified possible proteins incorporated by biotinylated substrate peptides and pentylamine for each TG isozyme and pan TGs, respectively. Further analyses of the identified unique substrates related to renal disease and fibrosis are important in order to understand the detailed mechanisms of disease progression and also to develop new drugs for renal disease treatment and antifibrotic therapies.

## Methods

### Materials

Chemical reagents were purchased from WAKO chemicals (Osaka, Japan) and Nacalai Tesk (Kyoto, Japan). Rabbit polyclonal anti-collagen I antibody was purchased from Abcam (Cambridge, UK). Rabbit polyclonal anti-TG1 and -TG2 sera were made by Japan Lamb (Hiroshima, Japan)^22^. Horseradish peroxidase and Alexa 594-conjugated anti-rabbit IgG were obtained from Jackson ImmunoResearch Laboratories (West Grove, PA, USA) or Cosmo Bio (Tokyo, Japan), and Invitrogen (Carlsbad, CA, USA), respectively. The 5-(biotinamido) pentylamine (BPA), a biotinylated primary amine substrate for the TGs, was obtained from Pierce (IL, USA), while 4′,6-diamidino-2-phenylindole (DAPI) was obtained from Sigma-Aldrich (St. Louis, MO, USA). Human tubular epithelial cells (HK-2) were purchased from ATCC (Manassas, VA, USA).

### Ethics statement

Animal experiments were conducted at Nagoya University, complying with the national guidelines for the care and use of laboratory animal. All animal experiments were approved by the animal care and use committee of Nagoya University. All surgeries were performed under anesthesia and all efforts were made to minimize suffering.

### Animal surgery and experimental protocol

ICR and C57BL/6J mice (8–10 weeks old) were purchased from Japan SLC Inc (Shizuoka, Japan) and group-housed with food and water available *ad libitum*. The surgical laparotomy and unilateral ureteral obstruction (UUO) was performed according to the method described by Shweke *et al*.^16^. Briefly, under the anesthesia after intraperitoneal injection of pentobarbital sodium, the left ureter was ligated at two separated points. Mice on days 0, 3, 7, and 14 after UUO surgery were perfused with PBS to remove the blood in kidney, and pieces of the kidney were either fixed in 4% paraformaldehyde for histological examination or frozen immediately in liquid nitrogen and stored for use in other experiments.

### Immunohistochemical analysis

Cryosections from the kidney (10 μm) were fixed, treated with 3% H_2_O_2_, incubated with blocking solution (diluted goat serum), and stained with TG1 and TG2 antibodies. Staining signals were enhanced using a VECTASTAIN ABC kit and developed with ImmPACT DAB (Vector Laboratories, Burlingame, CA, USA). As a negative control, the primary antibody was replaced with the same amount of rabbit non-immune IgG (NI-IgG) from Sigma-Aldrich. Sections were also stained with hematoxylin and eosin (Leica Microsystems, Wetzlar, Germany).

### Sirius red/fast green staining

Collagen fibers were detected using a Sirius Red Collagen Detection Kit (Chondrex, Redmond, USA). Collagen fibers appeared red, while the non-collagen proteins were green. The quantitative estimations of Sirius red staining were performed. Briefly, in the sections from each animal, more than 5 randomly selected microscopic fields were captured by a Keyence BZ-9000 microscope. All images were quantitatively estimated for collagen fibers in Sirius red staining within the respective kidney area according to the tutorial about “quantifying stained tissue” in image analyzer (Image J software, NIH). Each red color image was split as grayscale images and thresholded optimally. The positive areas above threshold level were measured and an average of at least 4 field from three replicates in each sample group was determined.

### Measurement of hydroxyproline contents

The hydroxyproline (HDP) contents were measured as described by Reddy *et al*.^54^. Briefly, approximately 30 mg of frozen kidney tissue was hydrolyzed in 2 N NaOH at 65 °C for 10 min, and then incubated at 120 °C for 20 min. Following this, the same amount of 6 N HCl was added and the mixture was then incubated at 120 °C for 20 min. This was then mixed with activated charcoal solution (10 mg/ml in 4 N KOH) and a four-fold concentration of acetate-citrate buffer (pH 6.5) containing 1.8 M sodium acetate, 0.5 M citric acid, 0.4 M acetic acid, and 1.7 M sodium hydroxide. After centrifugation, the supernatant was incubated with 100 mM chloramine T solution at room temperature for 25 min, after which 1 M Ehrlich’s solution was added and the samples incubated at 65 °C for 20 min. The absorbance of the samples was then measured at 560 nm. Data are provided as the mean ± standard deviation.

### Reverse transcription-polymerase chain reaction (RT-PCR)

Total RNA was extracted from the frozen kidneys and used for transcription by reverse-transcriptase (TAKARA Bio, Kyoto, Japan). The cDNAs as template were mixed with specific primer pairs (summarized in Table 3), following which PCR was performed. The amplified products were analyzed by 2.5% agarose gel electrophoresis.

**Table 3:** Primer pairs for RT-PCR experiments using mouse renal tissue.

Gene	Forward	Reverse	Product size
*Col1A1*	GAGCGGAGAGTACTGGATCG	TACTCGAACGGGAATCCATC	204
*α-SMA*	ACTGGGACGACATGGAAAAG	AGAGGCATAGAGGGACAGCA	203
*TGFβ1*	ATACGCCTGAGTGGCTGTCT	GGTTCATGTCATGGATGGTG	192
*FXIIIA*	TGATTGTCCGCAGAGGGCAG	GGGTAGCGACCAATGAC	105
*TG1*	ATCGTGGTAGTAGCCGACGC	ATGGTCACAGAGTCCGAGGC	138
*TG2*	AGCCGATGATGTGTACCTAG	AGGATTCCATCCTCGAACTG	137
*TG3*	TGGAGAAAGGCAGTGATAG	ACTGGAACCTTCTGGATAC	382
*TG4*	AGTCTGGCGTAGAGGTTATTC	CCTGAGCACCACGGATTG	125
*TG5*	GTTCCATTCTGGCAGGACAC	CCCAGGGCACTGATGCGGAT	133
*TG6*	GCTCTGTGCTTGACCAACCT	TGGGATTCACGCAGGATCTC	112
*TG7*	ACAGGGCAGTTCATTCTGGT	TGGTGAGGGTGATGTGGATA	117
*GAPDH*	ACTGGCATGGCCTTCCGTGT	CCTGCTTCACCACCTTCTTG	109

In the experiment using HK-2, quantitative RT-PCR was performed. Total RNA was isolated form cells using the Favorprep RNA mini kit (Favorgen, Biotech Corp., Denmark). Corresponding cDNA were prepared using ReverTra Ace qPCR RT Master Mix with gDNA Remover kit (TOYOBO, Osaka, Japan) and Real-time PCR analysis was performed using THUNDERBIRD SYBR qPCR Mix (TOYOBO) in an Eco real-time PCR system (Illumina, San Diego, CA, USA). Used specific primer pairs were summarized in Table 4.

**Table 4:** Primer pairs for quantitative RT-PCR experiments using human tubular epithelial cell.

Gene	Forward	Reverse	Product size
*Col1A1*	CCTGCGTGTACCCCACTCA	ACCAGACATGCCTCTTGTCCTT	83
*E-cad*	GAACGCATTGCCACATACAC	ATTCGGGCTTGTTGTCATTC	118
*TG1*	TGCCCAGAGGACATTGTGTA	GTGGTCAAACTGGCCGTAGT	135
*TG2*	ATGCCGACGTGGTAGACTGG	CACTGCCCATGTTCATGCTC	270
*GAPDH*	ACTGGCATGGCCTTCCGTGT	CCTGCTTCACCACCTTCTTG	109

### Western blot analysis

The mouse kidneys were homogenized in lysis buffer containing 10 mM Tris-HCl (pH 8.0), 150 mM NaCl, 0.1 mM EDTA, 0.1% Triton X-100, and protease inhibitor cocktail (Merck Millipore, Darmstadt, Germany). After centrifugation, the supernatant was obtained by treatment with SDS-containing buffer and boiling. For immunoblotting, samples were subjected to SDS-PAGE and transferred to a polyvinylidene difluoride (PVDF) membrane (Merck Millipore). After blocking with PBS containing 5% skim milk, the membrane was reacted with primary antibody, and the specific signal was detected by the secondary antibody conjugated with peroxidase and chemiluminesence reagent (Thermo Scientific, IL, USA). Each experiment was conducted in triplicate.

### *In situ* detection of TG activities

The *in situ* TG activity was visualized using FITC-labeled peptides from the unfixed kidney sections as reported previously^21^. The frozen specimen block was cut into 10-μm-thick sections using cryomicrotome (Leica Microsystems). These sections were collected using cryofilm, and then incubated in a reaction mixture containing 100 mM Tris-HCl (pH 8.0), 1 mM DTT, and 5 mM CaCl_2_ in the presence of 5 μM FITC-labeled peptide at 37 °C for 1 h. Following this, the sections were washed and mounted using SCMM-R2 (Leica Microsystems) or Fluoromount/Plus (Cosmo Bio). The fluorescence signals were observed under a fluorescence microscope (BZ-9000; Keyence, Osaka, Japan). The signal intensities in the images were adjusted to maintain linearity using imaging software (Adobe Photoshop CS). Each experiment was conducted in triplicate.

### *In vitro* siRNA knockdown of TG1 and TG2 and treatment of TGF-β1

TG1 siRNA (sense 5′-ccaucaucggcaaguuucatt-3′ and antisense 5′-ugaaacuugccgaugauggtt-3′), TG2 siRNA (sense 5′-cccugaucguugggcugaatt-3′ and antisense 5′-uucagcccaacgaucagggtt-3′), and MISSON siRNA universal negative control #1 (SIC-001) were purchased from Sigma-Aldrich. Reverse transfection was performed following the vendor’s protocol for RNAiMAX (Invitrogen). Briefly, the siRNA duplex was diluted in Dulbecco’s Modified Eagle medium (Nakarai Tesq) to yield a concentration of 50 nM mixture. The lipofectamine reagent RNAiMAX was then mixed to medium containing the siRNA solution at a ratio of 1:100 (v/v) and aliquoted in culture dishes to incubate for 15 min at room temperature. Then, an appropriate number of cells were added to the culture dishes. Twenty-four hours later, medium was replaced with serum-free culture medium for 12 h, then cells were treated with vehicle or TGF-β1 (5 ng/ml) for 12 h.

### Detection and identification of candidate substrates

The detection and substrate identification were performed as reported previously^23^. Kidney extracts were incubated with each biotinylated substrate peptide (pepK5 and pepT26) and pentylamine (BPA), following the same procedure as outlined above for the *in vitro* detection of activity. Following the crosslinking reaction by the endogenous enzymes in the extracts, these samples were applied to SoftLink™ Soft Release Avidin Resin (Promega, WI, USA). The biotinylated proteins were then eluted with 500 μl of 5 mM biotin. The eluted sample was subjected to SDS-PAGE and then stained using Silver Staining Kit (Kanto Chemical, Tokyo, Japan). The same eluted sample was also blotted on a PVDF membrane. The biotinylated peptide-incorporated proteins were detected using peroxidase-conjugated streptavidin and a chemiluminescence reagent.

For identification of candidate substrates, the purified samples by avidin resin were precipitated with 10% TCA/acetone, following which the samples were dissolved in 8 M urea and mixed with 5 μl of 0.2% Max surfactant (Promega)/50 mM NH_4_HCO_3_ in a vortex mixer. Then, 14.6 μl of 50 mM NH_4_HCO_3_ and 1.25 μl of 0.1 M DTT were added, and the mixture was incubated at 56 °C for 20 min. For the alkylation of the samples, 1.5 μl of 0.3 M iodoacetamide was added and the samples were trypsinized in the presence of 0.01% Max surfactant. These samples were then fractionated using a reverse-phase Dina Nano-HPLC in a C18 column (KYA Technologies, Tokyo, Japan). Each fraction was mixed with α-cyano-4-hydroxycinnamic acid and spotted on MALDI plate. MALDI-TOF mass spectrometry was then performed using a 5800 Proteomics Analyzer (ABSCIEX, Tokyo, Japan). Mass spectrometry and tandem mass spectrometry (MS/MS) data for each peptide were analyzed using Protein pilot™ software (ABSCIEX).

### Statistical analyses

Quantitative data are expressed as the means plus the standard deviation of three replicates from at least three independent experiments. The statistical significance of differences was assessed using Student’s t-test and the values of P < 0.05 were considered to indicate statistical significance.

## Electronic supplementary material

Supplementary information
