# Supplementary material for: Isozyme-specific comprehensive characterization of transglutaminase-crosslinked substrates in kidney fibrosis
Source: Sci Rep. 2018 May 9;8:7306. doi: 10.1038/s41598-018-25674-4 (PMC5943318; doi:10.1038/s41598-018-25674-4)
Supplement: Supplementary file 1 — Supplementary information [file 41598_2018_25674_MOESM1_ESM.pdf]

Supplementary Information

Isozyme-specific comprehensive characterization of transglutaminase-crosslinked substrates in kidney fibrosis

Hideki Tatsukawa*, Risa Otsu, Yuji Tani, Ryosuke Wakita and Kiyotaka Hitomi

Suppl Fig. S1

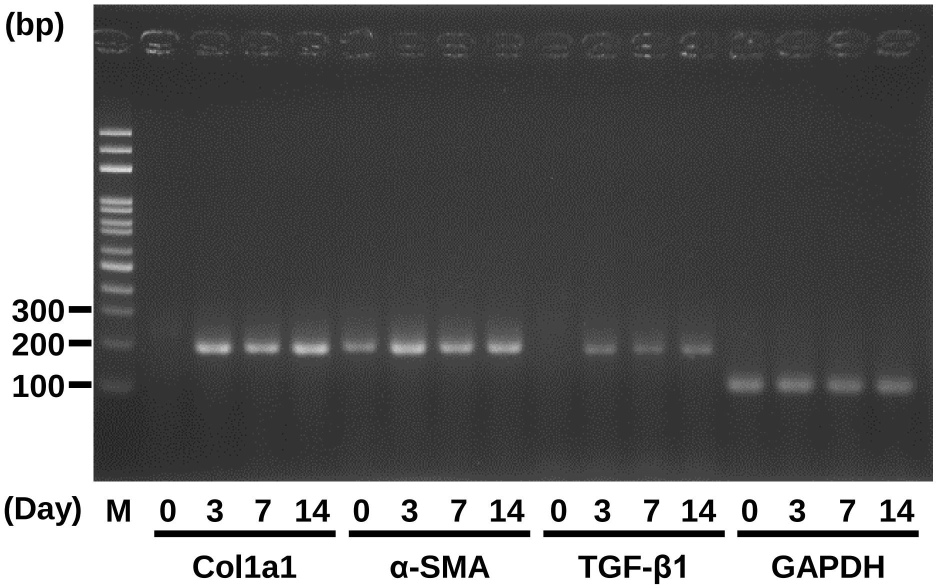

(bp)
300
200
100
(Day)
M
0 3 7 14 0 3 7 14 0 3 7 14 0 3 7 14
Col1a1
α-SMA
TGF-β1
GAPDH

Suppl Fig. S2

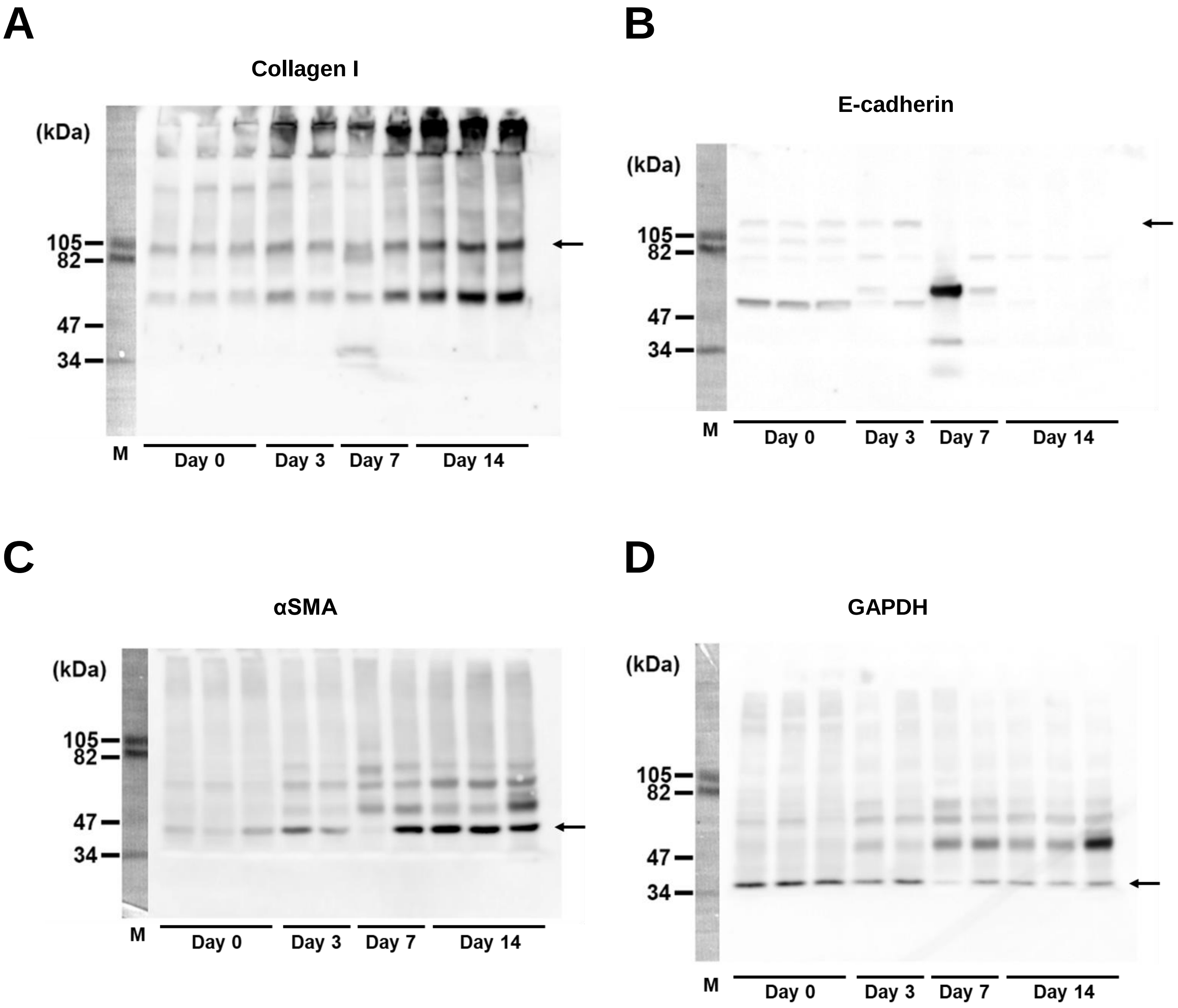
A
Collagen I
(kDa)
105
82
47
34
M
Day 0
Day 3
Day 7
Day 14
B
E-cadherin
(kDa)
105
82
47
34
M
Day 0
Day 3
Day 7
Day 14
C
αSMA
(kDa)
105
82
47
34
M
Day 0
Day 3
Day 7
Day 14
D
GAPDH
(kDa)
105
82
47
34
M
Day 0
Day 3
Day 7
Day 14

Suppl Fig. S3

A

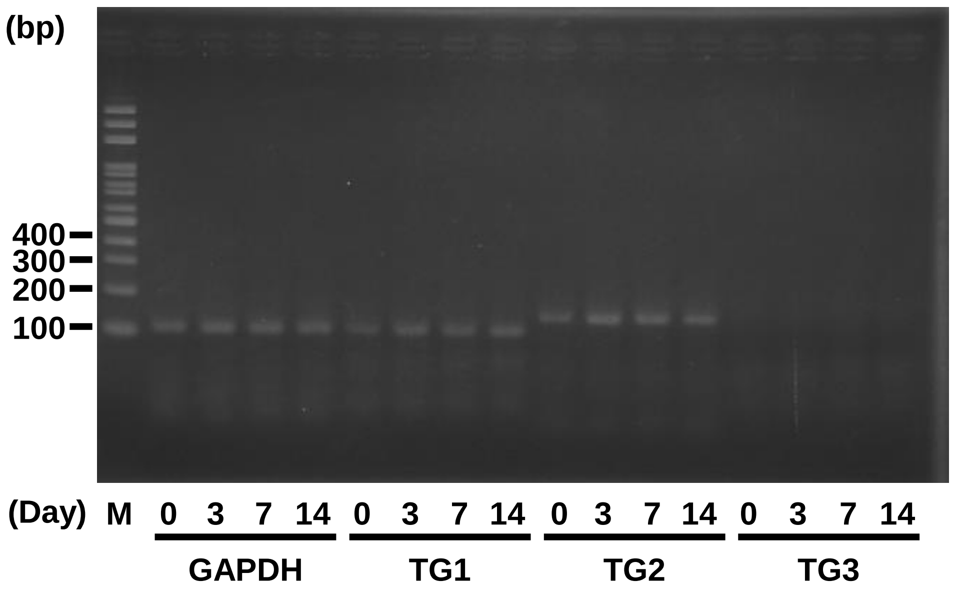

B

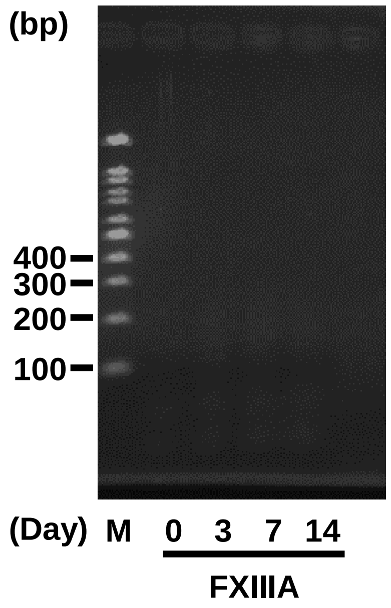

C

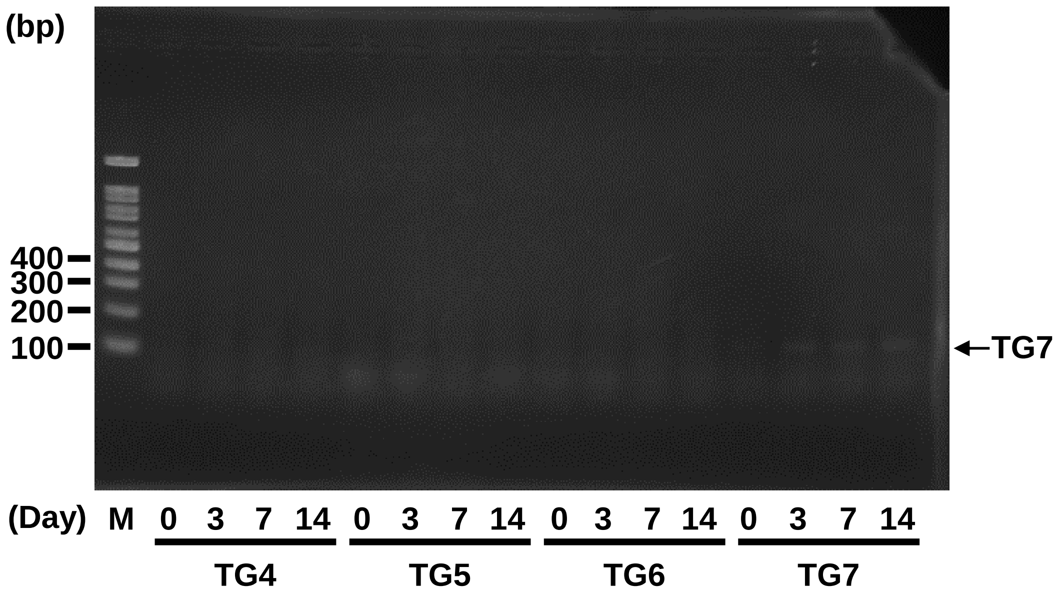

Suppl Fig. S4

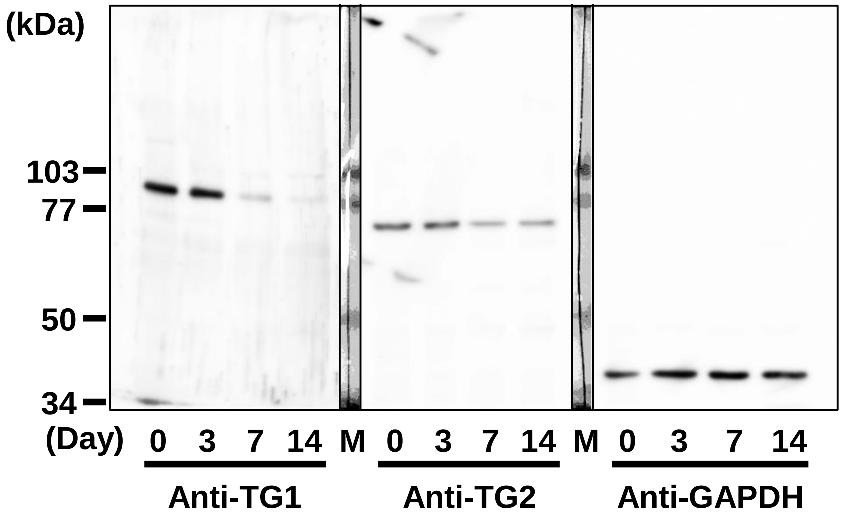
(kDa)
103
77
50
34
(Day)
0 3 7 14 M 0 3 7 14 M 0 3 7 14
Anti-TG1
Anti-TG2
Anti-GAPDH

Suppl Fig. S5

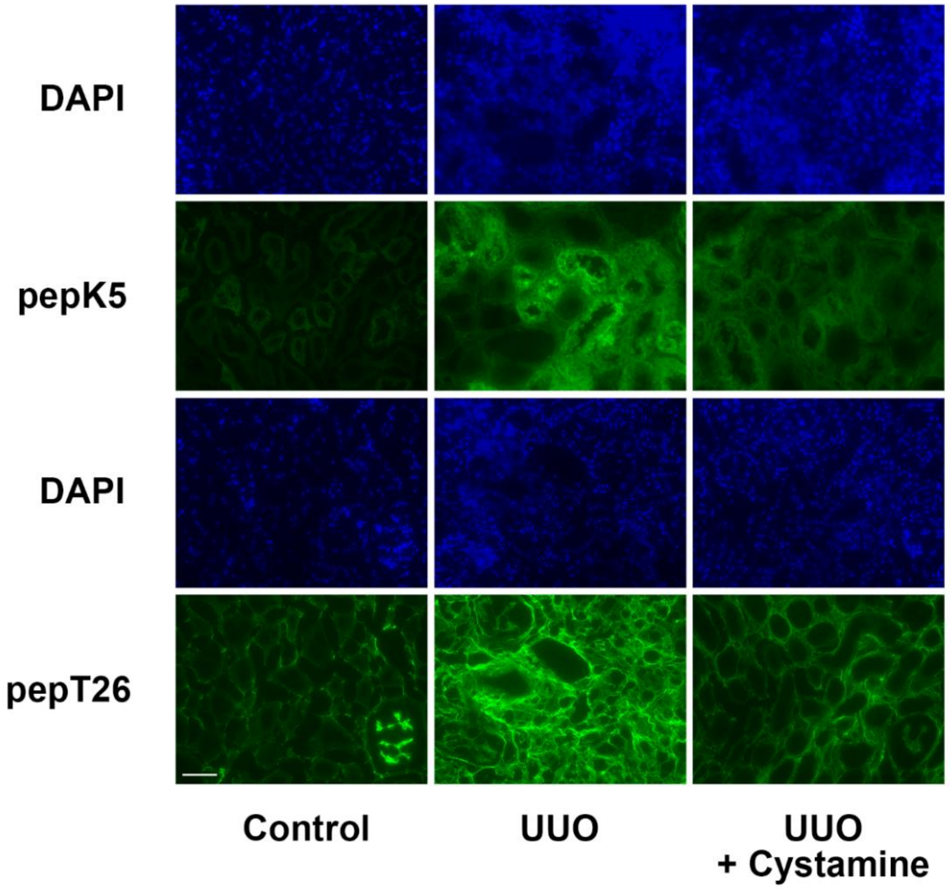

Suppl Fig. S6

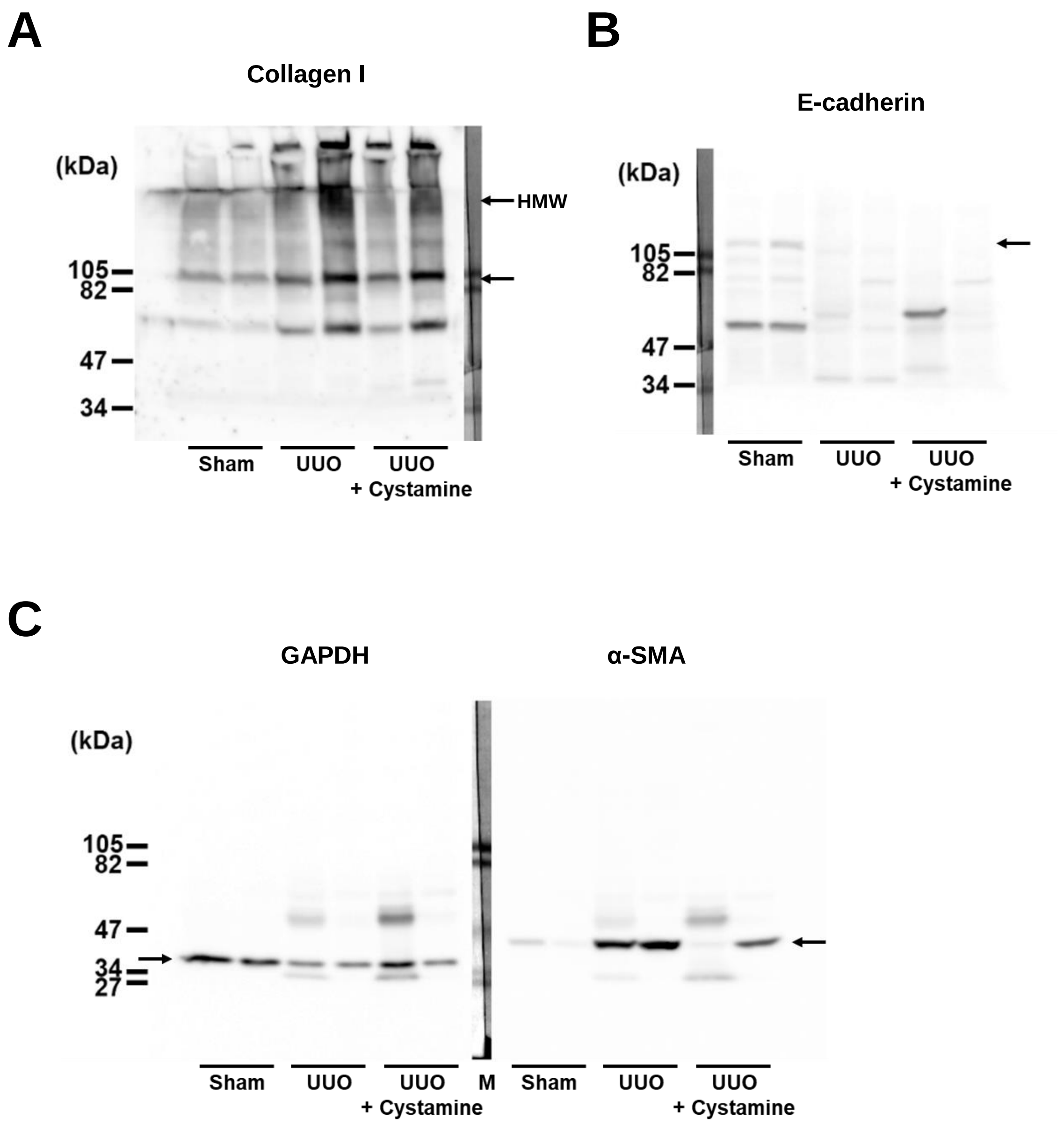
A
Collagen I
(kDa)
HMW
105
82
47
34
Sham
UUO
UUO
+ Cystamine
B
E-cadherin
(kDa)
105
82
47
34
Sham
UUO
UUO
+ Cystamine
C
GAPDH
α-SMA
(kDa)
105
82
47
34
27
Sham
UUO
UUO
+ Cystamine
M
Sham
UUO
UUO
+ Cystamine

Suppl Fig. S7

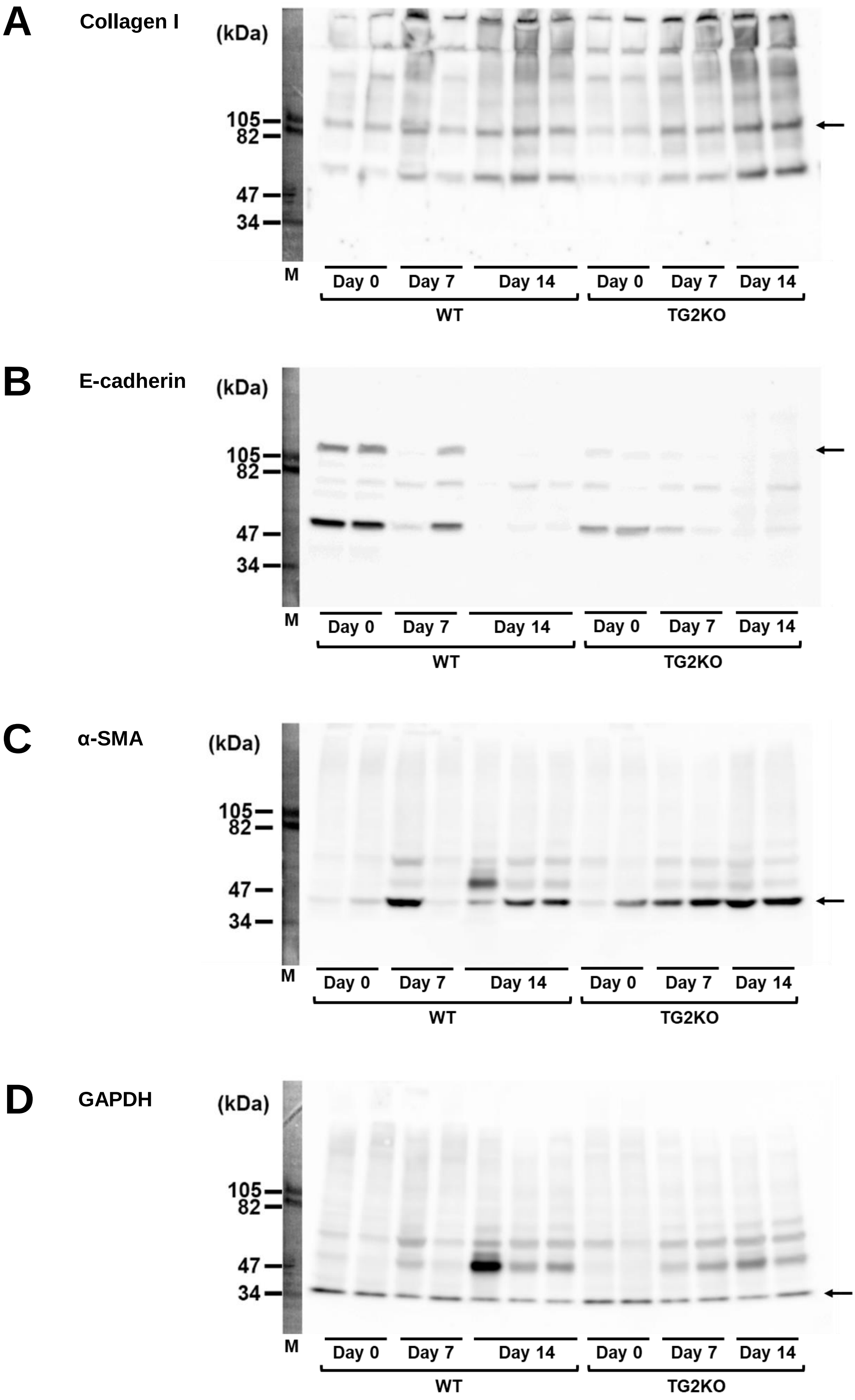

A
Collagen I
(kDa)
105
82
47
34
M
Day 0
Day 7
Day 14
Day 0
Day 7
Day 14
WT
TG2KO
B
E-cadherin
(kDa)
105
82
47
34
M
Day 0
Day 7
Day 14
Day 0
Day 7
Day 14
WT
TG2KO
C
α-SMA
(kDa)
105
82
47
34
M
Day 0
Day 7
Day 14
Day 0
Day 7
Day 14
WT
TG2KO
D
GAPDH
(kDa)
105
82
47
34
M
Day 0
Day 7
Day 14
Day 0
Day 7
Day 14
WT
TG2KO

Suppl Fig. S8

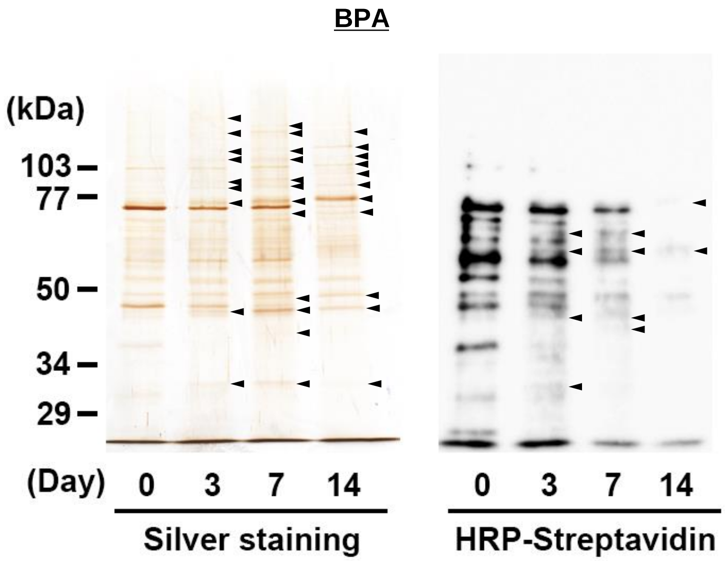

BPA
(kDa)
103
77
50
34
29
(Day)
0
3
7
14
Silver staining
0
3
7
14
HRP-Streptavidin

Suppl Fig. S9

A

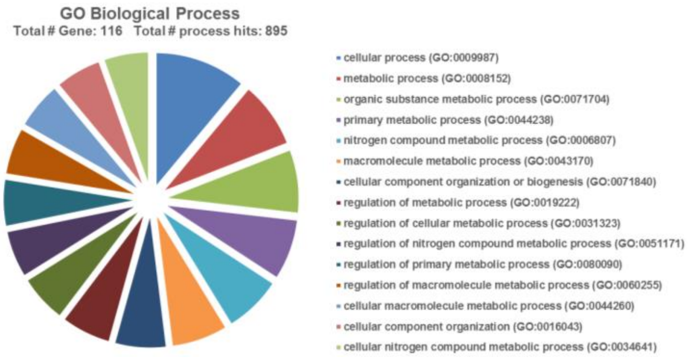

B

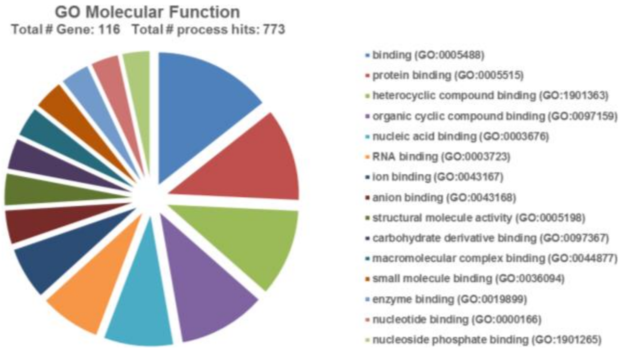

C

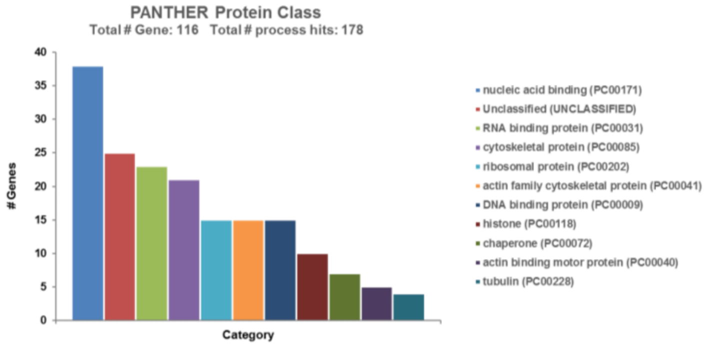

Supplementary Figure Legends

Supplementary Fig. S1 Example of full-length gel from Fig. 1C

The data of RT-PCR analysis in Fig. 1C have been cropped for presentation. The mRNA expression levels of the fibrotic markers (Collagen Iα1 (Col1a1), αSMA, TGF-β1, and GAPDH) were confirmed by RT-PCR.

Supplementary Fig. S2 Example of full-length blot from Fig. 1D

The data of western blot analysis in Fig. 1D have been cropped for presentation. The blots with whole lysates from the kidney tissue were cut at the center position in each marker lane and analyzed by immunoblotting using each indicated antibodies (anti-collagen I, E-cadherin, α-SMA, and GAPDH antibodies).

Supplementary Fig. S3 Example of full-length gels from Fig. 2A

The data of RT-PCR analysis in Fig. 2A have been cropped for being able to combine several data in one picture. The mRNA expression levels of the TG family (FXIIIA and TG1–7) were confirmed by RT-PCR (A-C). The successful detections for FXIIIA, TG3–6 using each primer pair are confirmed in the other tissue extracts.

Supplementary Fig. S4 Example of full-length blot from Fig. 2B

The data of western blot analysis in Fig. 2B have been cropped for presentation. The blots with whole lysates from the kidney tissue were cut at the center position in each marker lane and analyzed by immunoblotting using each indicated antibodies (anti-TG1, TG2, and GAPDH antibodies).

Supplementary Fig. S5 The effect of TGs inhibitor in the activities of TG1 and TG2

The *in situ* activities of TG1 and TG2 were visualized using FITC-labeled substrate peptides (pepK5 and pepT26, respectively) in the vehicle- or cystamine-treated mice under the UUO surgery. Bar = 50 μm.

Supplementary Fig. S6 Example of full-length blot from Fig. 5C

The data of western blot analysis in Fig. 5C have been cropped for presentation. The blots with whole lysates from the kidney tissue were cut at the center position in each marker lane and analyzed by immunoblotting using each indicated antibodies (anti-collagen I, E-cadherin, α-SMA, and GAPDH antibodies).

Supplementary Fig. S7 Example of full-length blot from Fig. 5I

The data of western blot analysis in Fig. 5I have been cropped for presentation. The blots with

whole lysates from the kidney tissue were cut at the center position in each marker lane and analyzed by immunoblotting using each indicated antibodies (anti-collagen I, E-cadherin, α-SMA, and GAPDH antibodies).

Supplementary Fig. S8 Detection of possible substrates incorporated with each peptide in kidney extracts

Each kidney extract on the indicated days after UUO surgery (n = 3 mice) was incubated with the biotinylated pentylamine (BPA). These proteins incorporated with BPA after the purification using monoavidin gel were subjected to silver staining and detected using peroxidase-conjugated streptavidin. The sizes of the protein mass markers are shown on the left. Arrowheads indicate the bands that increased compared with the control sample (Day 0).

Supplementary Fig. S9 Gene ontology (GO) terms for possible substrates for TGs during renal fibrosis

GO analysis corresponding to biological process (A), molecular function (B) and PANTHER protein class (C) represented as pie and bar charts generated by PANTHER classification system (http://www.pantherdb.org/).

Supplementary Table

Supplementary Table S1.

Accession number	Name
Q64433	10 kDa heat shock protein, mitochondrial
Q9CQ62	2,4-dienoyl-CoA reductase, mitochondrial
Q60597	2-oxoglutarate dehydrogenase, mitochondrial
O08756	3-hydroxyacyl-CoA dehydrogenase type-2
P47962	60S ribosomal protein L5
P20029	78 kDa glucose-regulated protein
Q8QZT1	Acetyl-CoA acetyltransferase, mitochondrial
Q99KI0	Aconitate hydratase, mitochondrial
P63260	Actin, cytoplasmic 2
P51881	ADP/ATP translocase 2
P55302	α-2-macroglobulin receptor-associated protein
P16460	Argininosuccinate synthase
Q03265	ATP synthase subunit α, mitochondrial
P56480	ATP synthase subunit β, mitochondrial
O35143	ATPase inhibitor, mitochondrial
P24270	Catalase
Q9CZU6	Citrate synthase, mitochondrial
P56391	Cytochrome c oxidase subunit 6B1
P62897	Cytochrome c, somatic
Q64462	Cytochrome P450 4B1
Q99LB2	Dehydrogenase/reductase SDR family member 4
Q9DCW4	Electron transfer flavoprotein subunit β
P26040	Ezrin
P05064	Fructose-bisphosphate aldolase A
Q91Y97	Fructose-bisphosphate aldolase B
P26443	Glutamate dehydrogenase 1, mitochondrial
P16858	Glyceraldehyde-3-phosphate dehydrogenase
Q9DCY0	Glycine N-acyltransferase-like protein Keg1
P63017	Heat shock cognate 71 kDa protein
P63158	High mobility group protein B1

**P62806**	**Histone H4**
**P54071**	**Isocitrate dehydrogenase [NADP], mitochondrial**
**Q9D646**	**Keratin, type I cuticular Ha4**
**Q61781**	**Keratin, type I cytoskeletal 14**
**Q9Z2K1**	**Keratin, type I cytoskeletal 16**
**Q9QWL7**	**Keratin, type I cytoskeletal 17**
**Q6IFX2**	**Keratin, type I cytoskeletal 42**
**P45952**	**Medium-chain specific acyl-CoA dehydrogenase, mitochondrial**
**P24369**	**Peptidyl-prolyl cis-trans isomerase B**
**P35700**	**Peroxiredoxin-1**
**Q9R0H0**	**Peroxisomal acyl-coenzyme A oxidase 1**
**Q9DBM2**	**Peroxisomal bifunctional enzyme**
**P51660**	**Peroxisomal multifunctional enzyme type 2**
**Q99MZ7**	**Peroxisomal trans-2-enoyl-CoA reductase**
**Q9WVE8**	**Protein kinase C and casein kinase substrate in neurons protein 2**
**Q05920**	**Pyruvate carboxylase, mitochondrial**
**Q8VDN2**	**Sodium/potassium-transporting ATPase subunit α-1**
**P14094**	**Sodium/potassium-transporting ATPase subunit β-1**
**P52592**	**Sphingosine 1-phosphate receptor 2**
**P38647**	**Stress-70 protein, mitochondrial**
**Q9CW03**	**Structural maintenance of chromosomes protein 3**
**Q9WUM5**	**Succinyl-CoA ligase [GDP-forming] subunit α, mitochondrial**
**Q9JJZ2**	**Tubulin α-8 chain**
**Q9D6F9**	**Tubulin β-4 chain**
**P50544**	**Very long-chain specific acyl-CoA dehydrogenase, mitochondrial**

Supplementary Table S1. Identified possible substrates for TG1 in control sample

Kidney extract on control sample (Day 0) was incubated with pepK5. The peptide-incorporated proteins were then purified using monoavidin gel and subjected to trypsin digestion. The fragmented peptides were fractionated by nano-HPLC and identified using MALDI-TOF/TOF mass spectrometer.

Supplementary Table S2.

Accession number	Name
Q9CQ62	2,4-dienoyl-CoA reductase, mitochondrial
P47962	60S ribosomal protein L5
Q8QZT1	Acetyl-CoA acetyltransferase, mitochondrial
P63260	Actin, cytoplasmic 2
P51881	ADP/ATP translocase 2
P24270	Catalase
Q8CGP7	Histone H2A type 1-K
Q8CGP1	Histone H2B type 1-K
P62806	Histone H4
Q61765	Keratin, type I cuticular Ha1
Q8K0Y2	Keratin, type I cuticular Ha3-I
Q61897	Keratin, type I cuticular Ha3-II
Q497I4	Keratin, type I cuticular Ha5
Q9Z2K1	Keratin, type I cytoskeletal 16
Q6IFX2	Keratin, type I cytoskeletal 42
Q99M74	Keratin, type II cuticular Hb2
Q9Z2T6	Keratin, type II cuticular Hb5
Q922U2	Keratin, type II cytoskeletal 5
P24369	Peptidyl-prolyl cis-trans isomerase B
Q9R0H0	Peroxisomal acyl-coenzyme A oxidase 1
Q9DBM2	Peroxisomal bifunctional enzyme
Q05920	Pyruvate carboxylase, mitochondrial
Q80VD1	REVERSED Protein FAM98B
P07724	Serum albumin
Q8VDN2	Sodium/potassium-transporting ATPase subunit α-1
P14094	Sodium/potassium-transporting ATPase subunit β-1
P52592	Sphingosine 1-phosphate receptor 2

Supplementary Table S2. Identified possible substrates for TG2 in control sample

Kidney extract on control sample (Day 0) was incubated with pepT26. The peptide-incorporated proteins were then purified using monoavidin gel and subjected to trypsin digestion. The fragmented peptides were fractionated by nano-HPLC and identified using MALDI-TOF/TOF mass spectrometer.

Supplementary Table S3.

Accession number	Name	Days			
		0	3	7	14
P20029	78 kDa glucose-regulated protein		+	+	+
P60710	Actin, cytoplasmic 1		+	+	+
P20060	Beta-hexosaminidase subunit β		+	+	+
P01029	Complement C4-B		+	+	+
P06909	Complement factor H		+	+	+
P11499	Heat shock protein HSP 90-β		+	+	+
Q9Z2X1	Heterogeneous nuclear ribonucleoprotein F		+	+	+
Q8VEK3	Heterogeneous nuclear ribonucleoprotein U		+	+	+
P30681	High mobility group protein B2		+	+	+
Q8CGP1	Histone H2B type 1-K		+	+	+
Q61781	Keratin, type I cytoskeletal 14		+	+	+
P08071	Lactotransferrin		+	+	+
O89017	Legumain		+	+	+
Q3THE2	Myosin regulatory light chain 12B		+	+	+
Q8VDD5	Myosin-9		+	+	+
Q61656	Probable ATP-dependent RNA helicase DDX5		+	+	+
Q921I1	Serotransferrin		+	+	+
P07724	Serum albumin		+	+	+
P42932	T-complex protein 1 subunit θ		+	+	+
P99024	Tubulin β-5 chain		+	+	+
P62702	40S ribosomal protein S4, X isoform		+	+	
Q9D8E6	60S ribosomal protein L4		+	+	
P47911	60S ribosomal protein L6		+	+	
P47738	Aldehyde dehydrogenase, mitochondrial		+	+	
P28352	DNA-(apurinic or apyrimidinic site) lyase		+	+	
P10126	Elongation factor 1-α 1		+	+	
P02104	Hemoglobin subunit ε-Y2		+	+	
Q99020	Heterogeneous nuclear ribonucleoprotein A/B		+	+	
O54879	High mobility group protein B3		+	+	
P84244	Histone H3.3		+	+	
P62960	Nuclease-sensitive element-binding protein 1		+	+	
Q61937	Nucleophosmin		+	+	
Q62446	Peptidyl-prolyl cis-trans isomerase FKBP3		+	+	

P05213	Tubulin α-1B chain	+	+	
Q6ZWV3	60S ribosomal protein L10	+		+
P63268	Actin, γ-enteric smooth muscle		+	+
P11087	Collagen α-1(I) chain		+	+
Q60847	Collagen α-1(XII) chain		+	+
P11276	Fibronectin		+	+
P14602	Heat shock protein beta-1		+	+
P26041	Moesin		+	+
Q60605	Myosin light polypeptide 6		+	+
P31725	Protein S100-A9		+	+
P52592	Sphingosine 1-phosphate receptor 2		+	+
P84104	Splicing factor, arginine/serine-rich 3		+	+
P26039	Talin-1		+	+
Q9CWF2	Tubulin β-2B chain		+	+
Q91X17	Uromodulin		+	+
Q8BWT1	3-ketoacyl-CoA thiolase, mitochondrial	+		
P62849	40S ribosomal protein S24	+		
P47962	60S ribosomal protein L5	+		
Q8K0E8	Fibrinogen β chain	+		
P07901	Heat shock protein HSP 90-alpha	+		
O35737	Heterogeneous nuclear ribonucleoprotein H	+		
Q8BFU2	Histone H2A type 3	+		
Q8C8R3-2	Isoform 2 of Ankyrin-2	+		
P35700	Peroxiredoxin-1	+		
P26043	Radixin	+		
Q8BMS1	Trifunctional enzyme subunit α, mitochondrial	+		
P68368	Tubulin α-4A chain	+		
P14131	40S ribosomal protein S16		+	
P97351	40S ribosomal protein S3a		+	
P62242	40S ribosomal protein S8		+	
O08848	60 kDa SS-A/Ro ribonucleoprotein		+	
P47963	60S ribosomal protein L13		+	
Q9CZM2	60S ribosomal protein L15		+	
Q9CPR4	60S ribosomal protein L17		+	
P35980	60S ribosomal protein L18		+	
P62717	60S ribosomal protein L18a		+	
Q8BP67	60S ribosomal protein L24		+	
P61255	60S ribosomal protein L26		+	

P14148	60S ribosomal protein L7	+	
P62737	Actin, aortic smooth muscle	+	
Q9WV32	Actin-related protein 2/3 complex subunit 1B	+	
P21550	β-enolase	+	
P29416	Beta-hexosaminidase subunit α	+	
Q6WVG3	BTB/POZ domain-containing protein KCTD12	+	
P30999	Catenin delta-1	+	
Q68FD5	Clathrin heavy chain 1	+	
P39061	Collagen α-1(XVIII) chain	+	
Q8BTM8	Filamin-A	+	
Q8BG05	Heterogeneous nuclear ribonucleoprotein A3	+	
P61979	Heterogeneous nuclear ribonucleoprotein K	+	
P10922	Histone H1.0	+	
Q3THW5	Histone H2A.V	+	
Q6ZWY9	Histone H2B type 1-C/E/G	+	
Q9D6R2	Isocitrate dehydrogenase [NAD] subunit α, mitochondrial	+	
O08677	Kininogen-1	+	
Q922Q8	Leucine-rich repeat-containing protein 59	+	
Q61879	Myosin-10	+	
Q9WTI7	Myosin-Ic	+	
Q5SYD0	Myosin-Id	+	
Q64331	Myosin-VI	+	
Q9CY58	Plasminogen activator inhibitor 1 RNA-binding protein	+	
P17225	Polypyrimidine tract-binding protein 1	+	
Q501J6	Probable ATP-dependent RNA helicase DDX17	+	
Q01405	Protein transport protein Sec23A	+	
Q9D662	Protein transport protein Sec23B	+	
P52480	Pyruvate kinase isozymes M1/M2	+	
Q8BL97	Splicing factor, arginine/serine-rich 7	+	
P11983	T-complex protein 1 subunit α	+	
P42669	Transcriptional activator protein Pur-α	+	
O35295	Transcriptional activator protein Pur-β	+	
P50516	V-type proton ATPase catalytic subunit A	+	
Q9CZX8	40S ribosomal protein S19		+
P28653	Biglycan		+
Q61147	Ceruloplasmin		+

**P13020**	**Gelsolin**	**+**
**Q6GSS7**	**Histone H2A type 2-A**	**+**
**Q9ET77**	**Junctophilin-3**	**+**
**P28825**	**Meprin A subunit α**	**+**
**P11247**	**Myeloperoxidase**	**+**
**O09043**	**Napsin-A**	**+**
**Q8VCI0**	**Putative phospholipase B-like 1**	**+**
**Q7TNC4**	**Putative RNA-binding protein Luc7-like 2**	**+**
**Q80VD1**	**REVERSED Protein FAM98B**	**+**

Supplementary Table S3. Identified possible substrates for TGs using BPA

Kidney extract on each indicated day after UUO surgery was incubated with BPA. The newly identified possible substrates in each indicated day were demonstrated as "+" compared to control sample (Day 0).

Supplementary Table S4.

Accession number	Name
Q9CQ62	2,4-dienoyl-CoA reductase, mitochondrial
Q60597	2-oxoglutarate dehydrogenase, mitochondrial
O08756	3-hydroxyacyl-CoA dehydrogenase type-2
P62270	40S ribosomal protein S18
Q8QZT1	Acetyl-CoA acetyltransferase, mitochondrial
P63260	Actin, cytoplasmic 2
P51881	ADP/ATP translocase 2
P55302	α-2-macroglobulin receptor-associated protein
Q8C8R3	Ankyrin-2
P16460	Argininosuccinate synthase
Q03265	ATP synthase subunit α, mitochondrial
P56480	ATP synthase subunit β, mitochondrial
O35143	ATPase inhibitor, mitochondrial
P24270	Catalase
Q9CZU6	Citrate synthase, mitochondrial
Q9QZQ8	Core histone macro-H2A.1
Q9QZQ8	Core histone macro-H2A.1
P56391	Cytochrome c oxidase subunit 6B1
P62897	Cytochrome c, somatic
Q99LB2	Dehydrogenase/reductase SDR family member 4
Q8CHT0	Delta-1-pyrroline-5-carboxylate dehydrogenase, mitochondrial
Q9D2G2	Dihydrolipoyllysine-residue succinyltransferase component of 2-oxoglutarate dehydrogenase complex, mitochondrial
Q9DCW4	Electron transfer flavoprotein subunit β
P26040	Ezrin
P05064	Fructose-bisphosphate aldolase A
Q91Y97	Fructose-bisphosphate aldolase B
P26443	Glutamate dehydrogenase 1, mitochondrial
P16858	Glyceraldehyde-3-phosphate dehydrogenase
Q9DCY0	Glycine N-acyltransferase-like protein Keg1
P63017	Heat shock cognate 71 kDa protein
P01942	Hemoglobin subunit α
P63158	High mobility group protein B1
Q8R1M2	Histone H2A.J

P27661	Histone H2A.x
Q8CGP2	Histone H2B type 1-P
Q9D2U9	Histone H2B type 3-A
P84228	Histone H3.2
P62806	Histone H4
P62806	Histone H4
O88844	Isocitrate dehydrogenase [NADP] cytoplasmic
P54071	Isocitrate dehydrogenase [NADP], mitochondrial
Q6IFX2	Keratin, type I cytoskeletal 42
Q9Z2T6	Keratin, type II cuticular Hb5
Q922U2	Keratin, type II cytoskeletal 5
P45952	Medium-chain specific acyl-CoA dehydrogenase, mitochondrial
Q62425	NADH dehydrogenase [ubiquinone] 1 α subcomplex subunit 4
P24369	Peptidyl-prolyl cis-trans isomerase B
Q9R0H0	Peroxisomal acyl-coenzyme A oxidase 1
Q9DBM2	Peroxisomal bifunctional enzyme
P51660	Peroxisomal multifunctional enzyme type 2
Q99MZ7	Peroxisomal trans-2-enoyl-CoA reductase
Q9WUA2	Phenylalanyl-tRNA synthetase β chain
Q7TNG8	Probable D-lactate dehydrogenase, mitochondrial
Q9WVE8	Protein kinase C and casein kinase substrate in neurons protein 2
Q05920	Pyruvate carboxylase, mitochondrial
P35486	Pyruvate dehydrogenase E1 component subunit α, somatic form, mitochondrial
Q3UNZ8	Quinone oxidoreductase-like protein 2
Q8VDN2	Sodium/potassium-transporting ATPase subunit α-1
P14094	Sodium/potassium-transporting ATPase subunit β-1
Q78PY7	Staphylococcal nuclease domain-containing protein 1
P38647	Stress-70 protein, mitochondrial
Q9CW03	Structural maintenance of chromosomes protein 3
Q9WUM5	Succinyl-CoA ligase [GDP-forming] subunit α, mitochondrial
Q9D0R2	Threonyl-tRNA synthetase, cytoplasmic
P68373	Tubulin α-1C chain
P68372	Tubulin β-2C chain
P70691	UDP-glucuronosyltransferase 1-2
P50544	Very long-chain specific acyl-CoA dehydrogenase, mitochondrial

Supplementary Table S4. Identified possible substrates for TGs in control sample

Kidney extract on control sample (Day 0) was incubated with BPA. The peptide-incorporated proteins were then purified using monoavidin gel and subjected to trypsin digestion. The fragmented peptides were fractionated by nano-HPLC and identified using MALDI-TOF/TOF mass spectrometer.

Supplementary Table S5.

		ICR		
		Body Weight (mg)	Blood glucose (mg/dl)	Kidney weight (mg)
Day 7	Sham	37.2 ± 1.4	122.7 ± 18.5	-
	UUO	33.8 ± 2.2*	113.8 ± 16.1	-
	UUO + Cystamine	31.0 ± 3.0	100.5 ± 15.6	-
Day 14	Sham	38.7 ± 1.3	131.7 ± 20.0	312.2 ± 50.4
	UUO	35.4 ± 2.0*	106.5 ± 25.3	224.3 ± 38.0
	UUO + Cystamine	33.0 ± 3.4	100.8 ± 15.6	195.1 ± 17.7

Supplementary Table S5. Biological parameters in cystamine-treated mice after UUO surgery
Sham, sham-operated mice; *n* = 4 in mice per group. The data are presented as the mean ± SD (**P*<0.05 in mice as compared to sham mice, Student's t-test).

Supplementary Table S6.

		C57BL/6J		
		Body Weight (mg)	Blood glucose (mg/dl)	Kidney weight (mg)
Day 7	WT Sham	19.5 ± 3.6	158.8 ± 4.9	-
	WT UUO	18.9 ± 1.2	120.3 ± 18.0**	-
	TG2KO Sham	22.1 ± 4.1	197.3 ± 8.6††	-
	TG2KO UUO	20.2 ± 1.4	193.7 ± 65.0‡	-
Day 14	WT Sham	20.8 ± 1.0	148.3 ± 6.2	140.9 ± 23.1
	WT UUO	19.2 ± 1.5	81.7 ± 8.1**	172.9 ± 21.8
	TG2KO Sham	22.6 ± 1.0	154.0 ± 7.3	123.0 ± 9.7
	TG2KO UUO	20.9 ± 1.6	81.3 ± 9.5**	154.3 ± 15.8

Supplementary Table S6. Biological parameters in WT and TG2KO mice after UUO surgery
Sham, sham-operated mice; *n* = 4 in mice per group. The data are presented as the mean ± SD (***P*<0.01 in UUO-treated mice as compared to sham mice; ††*P*<0.01 in TG2KO mice as compared to corresponding WT mice; ‡*P*<0.05 in TG2KO mice after UUO surgery as compared to corresponding WT mice; Student's t-test).
